# Posaconazole: An Update of Its Clinical Use

**DOI:** 10.3390/pharmacy3040210

**Published:** 2015-10-21

**Authors:** Simon Leung, Mara N. Poulakos, Jade Machin

**Affiliations:** 1 Memorial Regional Hospital, Hollywood, FL 33021, USA; E-Mail: sleung@mhs.net; 2 Gregory School of Pharmacy, Palm Beach Atlantic University, West Palm Beach, FL 33416; USA; E-Mail: mara_poulakos@pba.edu; 3 Boca Raton Regional Hospital, Boca Raton, FL 33486, USA; E-Mail: jmachin@brrh.com

**Keywords:** posaconazole, pharmacokinetics, pharmacodynamics, *crytococcus neoformans*, *Candida*, *aspergillus*, fusariosi, neutropenia, candidiasis, graft-*versus*-host

## Abstract

Posaconazole (PCZ) is a relatively new addition to the azole antifungals. It has fungicidal activities against *Aspergillus fumigatus*, *Blastomyces dermatitidis*, selected *Candida* species, *Crytopcoccus neoformans*, and *Trichosporon*. PCZ also has fungistatic activities against *Candida*, *Coccidioides*, selected *Fusarium* spp., *Histoplasma*, *Scedosporium* and *Zygomycetes.* In addition, combining the drug with caspofungin or amphotericin B results in a synergistic interaction against *A. fumigatus*, *C. glabrata* and *C. neoformans*. The absorption of PCZ suspension is enhanced when given with food, nutritional supplements, and carbonated beverages. Oral administration of PCZ in divided doses also increases its bioavailability. PCZ has a large volume of distribution and is highly protein bound (>95%). The main elimination route of PCZ is fecal. PCZ is an inhibitor of the CYP3A4 enzyme; therefore, monitoring for drug-drug interactions is warranted with other CYP3A4 substrates/inhibitors/inducers. The most common adverse effects include headache, fatigue, nausea, vomiting and elevated hepatic enzymes. PCZ, with its unique antifungal activities, expands the azole class of antifungal agents. Because of its limit in formulation, PCZ oral suspension is recommended in immunocompromised patients with functional gastrointestinaltracts who fail conventional antifungal therapies or who are suspected to have a breakthrough fungal infection. However, a delayed-release tablet formulation and intravenous (IV) injection became available in 2014, expanding the use of PCZ in other patient populations, including individuals who are unable to take oral formulations.

## 1. Introduction

Posaconazole (PCZ) oral suspension (Noxafil^TM^, Schering-Plough Corporation) has received approval from the U.S. Food and Drug Administration (FDA) for prophylaxis against invasive aspergillosis and candidiasis in immunocompromised patients, 13 years of age and older [1,2]. PCZ is considered an extended-spectrum antifungal agent due to its unique spectrum of activity against various fungi, including the majority of yeasts, filamentous fungi and azole resistant *Candida* species (spp.) [3]. The patterns of *Candida* infections have shifted from the previous most common fluconazole (FCZ) sensitive *C. albicans* to other FCZ dose-dependent sensitive and FCZ resistant *Candida* species since the year 2000 [4]. These *Candida* species include, but are not limited to, *C*. *glabata*, *C. parapsilosis* and *C. tropicalis.* To complicate the treatment of fungal infections further, the emergence of breakthrough fungal infections (*i.e.*, *fusarium*, *scedosporium* and *zygomycosis*) has occurred secondary to the use of potent immunosuppressive agents and a lack of antifungal activity against these pathogens from current antifungals [5]. Therefore, novel additions to the antifungal armamentarium are needed. Because of its extended-spectrum against a broader range of pathogens, PCZ offers a promising role in treating refractory fungal infections. The focus of this article is to review and provide updated information on the unique characteristics of PCZ.

## 2. Chemical Structure

**Figure 1:**
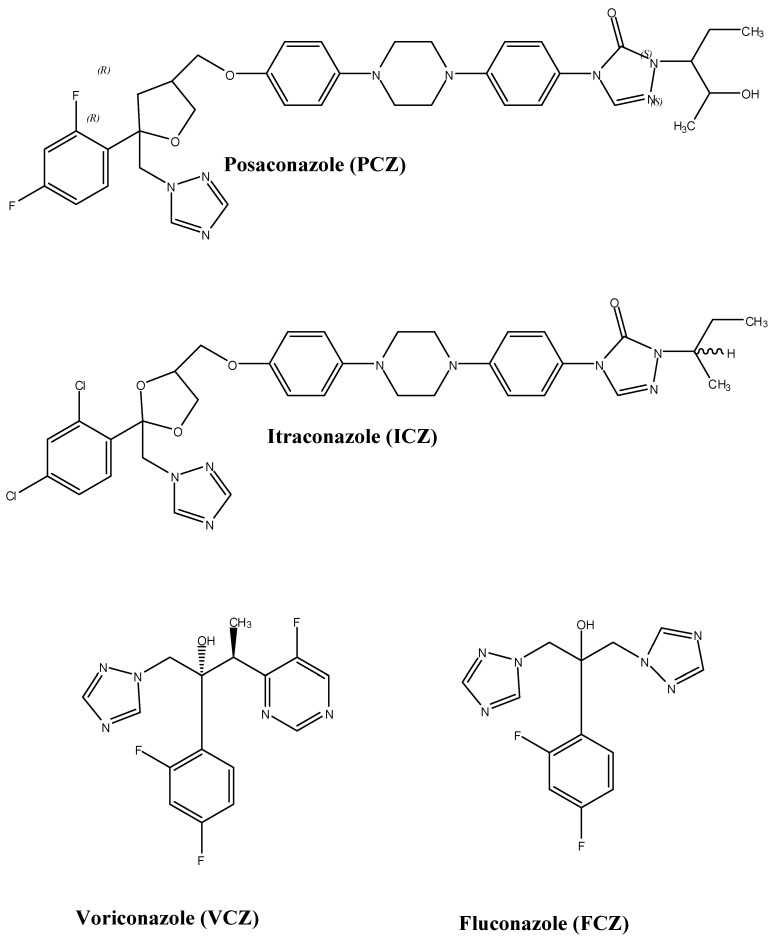
Structures of selected triazole antifungal agents.

PCZ (MW 700.8, pKa 3.6 and 4.6) is highly lipophilic with a triazole structure similar to itraconazole (ICZ) (Figure 1) [6]. The chemical structure of PCZ is an enantiomerically pure RRSS isomer with four chiral centers. PCZ’s structure consists of a classic triazole and difluoro phenyl rings with common orientations characteristic of other azoles. The tetrahydrofuran oxygen of PCZ overlaps the hydroxyl oxygen component of FCZ [7]. The long side chain of PCZ mounts securely inside the space formed by the various structural domains of lanosterol 14α-demethylase, a cytochrome P450 (CYP450) enzyme, which contains helix A′, a β turn connecting strands β4-1 and β4-2, an FG loop, helix B′ and β-strands, β1-4 [7].

## 3. Mechanism of Action

PCZ exerts its activity by inhibiting fungal ergosterol biosynthesis. PCZ binds to the heme cofactor situated on the target site of lanosterol 14α-demethylase, which is a CYP450 dependent enzyme [7,8]. This enzyme is the product of CYP51A gene expression (ERG11). The integrity of the fungal cell membrane is maintained by ergosterol. PCZ prevents demethylation of ergosterol at C-14 and/or C-4 positions. The altered structure of ergosterol interferes with plasma membrane function, resulting in fungal cell death.

Several *in vitro* studies have confirmed the antifungal mechanism of PCZ with liquid chromatography techniques [9,10]. Heimark *et al.* reported that *in vitro* PCZ depletes the production of ergosterol in *C. albicans* by inhibiting the enzyme lanosterol 14α-demethylase [9]. Similar studies have also demonstrated the depletion of ergosterol in both azole susceptible and resistant *C. albicans* when incubated with PCZ [minimum inhibitory concentrations, (MICs) ≤ 0.5 mcg/mL] [10]. Dose dependent inhibition of ergosterol and ergosterol-like compounds (*i.e.*, ergosta-5,8,22-trien-3-ol) was also observed in most *C. glabrata* strains and strain C110 respectively, when exposed to PCZ (MICs ≤ 4 mcg/mL). Exposure of *Aspergillus fumigatus* and *A. flavus* to PCZ (MICs ≤ 0.06 mcg/mL) also resulted in the inhibition of ergosterol synthesis.

## 4. Spectrum of Activity

PCZ has an extended spectrum of antifungal activity, as illustrated in Table 1 [11]. It has shown to have fungicidal activity *in vitro* and *in vivo*, against *Cryptococcus neoformans*, *Aspergillus (fumigatus*, *flavus*,and *terreus*), *Blastomyces dermatitidis*, *Trichosporon* and specific *Candida* spp. *(i.e.*, *C. krusei*, *C. parapsilosis*, *C. lusitaniae* and *C. inconspicua*) [11]. PCZ also has fungistatic activity against most other *Candida* spp., *Scedosporium*, *Coccidiodes*, *Zygomycetes* and specific *Fusarium* strains (*i.e.*, *F. oxysporum* and *F. monilforme)* [12]. However, unlike antibacterials, *in vitro* and *in vivo* activities of antifungal agents do not relate with clinical outcomes.

## 5. Pharmacodynamics

The *in vitro* activity of PCZ was compared with that of amphotericin B (AMB), FCZ and ICZ against isolates of *C. neoformans*. Yeasts were inhibited and killed at lower concentrations of PCZ (MICs, ranged from 0.063 to 0.25 mcg/mL) than those of AMB (MICs ranged from 0.25 to1 mcg/mL). Inhibition was also noted at concentrations at least ten fold lower than those of FCZ (MICs ranged from 0.5 to16 mcg/mL) but not for ICZ (MICs ranged from 0.008 to 0.031 mcg/mL). In the three isolates used, which were clinical strains of *C. neoformans* (T-1, DUMC 133.95, and 89–610), growth was inhibited at high MIC values of FCZ (8–16 mcg/mL) in contrast to low MIC values of PCZ (0.125 to 0.25 mcg/mL). The *in vivo* activities of PCZ when compared to FCZ were similar in terms of reducing yeast counts in the cerebrospinal fluid of a rabbit model [13]. Barchiesi *et al.* demonstrated that PCZ, when compared to AMB, was effective in prolonging survival of mice infected with *C. neoformans*. In fact, PCZ was superior to AMB at reducing fungal burden in the brains of mice infected by two *Cryptococcal* isolates [14].

An *in vitro* study of voriconazole (VCZ), FCZ and PCZ against isolates of *Candida* and *C. neoformans*, found that both VCZ and PCZ had greatest activity against *C. albicans*, *C. parapsilosis*, *C. krusei* and *C. lusitaniae*, *C. tropicalis*, *C. dubliniensis*, and *C. kefyr* [15]. Moreover, 97%–98% of *Candida* spp. were susceptible at MICs ≤ 1 mcg/mL. Both PCZ and VCZ were less active against *C*. *glabrata* (80% susceptible at MICs ≤ 1 mcg/mL) and PCZ was less active against *C. pelliculosa* (44% susceptible at MICs ≤ 1 mcg/mL). FCZ was most active (≥95% susceptible at MICs ≤ 8 mcg/mL) against *C. albicans* (99% susceptible), *C. parapsilosis* (95%), *C. lusitaniae* (98%), *C. tropicalis* (98%), and *C. pelliculosa* (100% susceptible) and least active against *C. glabrata* (57%) and *C. krusei* (1%). Both VCZ and PCZ were highly active against *C. neoformans* (98%–100% susceptible at MICs ≤ 1 mcg/mL) when compared to FCZ (98% of *C. neoformans* susceptible at MICs ≤ 8 mcg/mL) [15].

A clinical study compared the activities of PCZ, ICZ and FCZ against 3312 clinical isolates of *Candida* and 373 isolates of *C. neoformans*. PCZ was shown to be more active (97% of the *Candida* spp. and 100% of *C. neoformans* were inhibited at MIC ≤ 1 mcg/mL) than both triazole antifungals. Only 78% of *C. neoformans* isolates were inhibited by FCZ (MICs ≤ 8 mcg/mL) compared to 96% by PCZ and 68% by ICZ (MICs ≤ 0.25 mcg/mL). Furthermore, *C. albicans*, *C. parapsilosis*, *C. krusei*, *C. lusitaniae*, *C. tropicalis*, *C. dubliniensis*, *C. guilliermondii* and *C. famata* were found to be most susceptible to PCZ (99%–100% of isolates susceptible at MICs ≤ 1 mcg/mL) whereas *C*. *glabrata* was least susceptible (80% susceptible at MIC ≤ 1 mcg/mL) [16]. Barchiesi *et al.* compared the *in vitro* activities of FCZ, ICZ, PCZ, AMB and 5-fluorocytosine against 56 clinical isolates of *Candida*. All *Candida* strains were susceptible to PCZ (MIC range ≤ 0.007–0.125 mg/L) and AMB (MIC range ≤ 0.03–0.5 mg/L) compared to 97% and 95% of the isolates susceptible to FCZ (MIC range ≤ 0.125–32 mg/L) and ICZ (≤0.007–1 mg/L), respectively [17].

**Table 1:** Comparative *in vitro* activities of posaconazole (PCZ), itraconazole (ICZ), voriconazole (VCZ) and amphotericin B (AMB) against molds and yeasts collected from 200 medical centers worldwide over a 10-year period [11]

		PCZ		ICZ		VCZ		AMB	
MIC (mcg/mL)		50%	90%	50%	90%	50%	90%	50%	90%
**Organisms**	**n**								
All *Aspergillus* spp.	1423	0.125	0.5	0.5	2.0	0.25	0.5	0.5	1.0
*A. flavus*	89	0.25	0.5	0.5	1.0	0.5	1.0	1.0	2.0
*A. famigatus*	1119	0.125	0.5	0.5	1.0	0.25	0.5	0.5	1.0
*A. niger*	101	0.25	0.5	1.0	2.0	0.5	2.0	0.125	1.0
*A. terreus*	22	0.25	0.25	0.5	0.5	0.25	0.5	2.0	2.0
All *Zygomycetes*	86	0.5	4.0	1.0	32.0	16.0	128.0	0.25	2.0
*Rhizopus* spp.	32	1.0	8.0	4.0	32.0	16.0	128.0	1.0	2.0
*Mucor* spp.	18	1.0	16.0	2.0	32.0	64.0	128.0	0.25	1.0
*Absidia* spp.	16	0.125	0.25	0.125	0.5	16.0	128.0	0.25	0.5
*Cunninghamella* spp.	6	0.031–1	0.031–1	0.125–2	0.125–2	8–128	8–128	0.125–2	0.125–2
*Apophysomyces*	5	0.031–4	0.031–4	0.031–8	0.031–8	16–128	16–128	0.031–4	0.031–4
*Saksenaea* spp.	4	0.016–2	0.016–2	0.016–0.125	0.5–4	0.5–4	0.5–4	0.063–0.5	0.063–0.5
*Rhizomucor* spp.	3	0.016–0.25	0.016–0.25	0.016–0.25	0.016–0.25	2–16	2–16	0.063–0.125	0.063–0.125
*Cokeromyces* spp.	2	0.25–4	0.25–4	0.25–8	0.25–8	16–64	16–64	0.125–0.5	0.125–0.5
All *Fusarium* spp.	67	16	32	16	32	16	32	8.0	32
*F. solani*	39	32	32	ND	ND	16	32	16	32
*F. oxysporum*	12	2.0	4.0	ND	ND	4.0	32	8.0	16
*F. moniliforme*	2	1.0	1.0	ND	ND	1.0	1.0	1–4	1–4
Other *Fusarium* spp.	14	16	16	ND	ND	4.0	16.0	1.0	2.0
All *Candida* spp.	6965	0.063	1.0	0.125	1.0	0.031	0.5	1.0	1.0
*C. albicans*	3525	0.031	0.063	0.063	0.25	0.008	0.063	1.0	1.0
*C. glabrata*	1218	1.0	2.0	1.0	4.0	0.25	2.0	1.0	1.0
*C. parapsilosis*	970	0.063	0.25	0.5	0.5	0.031	0.125	1.0	1.0
*C. tropicalis*	719	0.063	0.125	0.5	0.5	0.063	0.5	1.0	1.0
*C. krusei*	189	0.5	1.0	1.0	1.0	0.25	0.5	1.0	2.0
*C. lusitaniae*	84	0.063	0.25	0.25	2.0	0.031	0.063	1.0	2.0
*C. guillermondii*	26	0.25	1.0	0.5	4.0	0.063	8.0	0.5	1.0
*C. dubliniensis*	164	0.031	0.125	0.063	0.5	0.016	0.125	0.5	1.0
Other *Candida*	60	0.25	2.0	0.5	1.0	0.063	0.25	1.0	1.0
Other Organisms									
*Cryptococcus* spp.	271	0.125	0.5	0.125	0.5	0.063	0.125	1.0	1.0
*Scedosporium prolificans*	80	16.0	32	64	64	ND	ND	16	32
*Scedosporium apiospermum*	26	0.25	1.0	1.0	32	ND	ND	2.0	8.0
*Coccidioides* spp.	25	0.125	0.25	0.125	0.25	ND	ND	0.5	0.5
*Blastomyces*	38	0.063	0.125	0.031	2.0	ND	ND	0.125	0.5
*Histoplasma*	53	0.019	0.25	0.019	0.063	ND	ND	0.25	0.5
*Pseudallescheria*	41	0.25	1.0	0.5	1.0	ND	ND	2.0	4.0

A 50% and 90% are minimum inhibitory concentrations (MIC) at which the growth of 50% and 90%, respectively, of the isolates were inhibited; b When *n* < 10, MIC ranges are given. ND, not determined.

## 6. *Cryptococcus neoformans*

An *in vitro* study by Barchiesi *et al.* investigated the effects of the interaction of PCZ and flucytosine (FC) against *C. neoformans* [18]. Synergy and additivism were observed in 33% and 67% of isolates, respectively. Indifference and antagonism were not observed. Even when synergy was not reached, the geometric mean MICs of both drugs dropped significantly when they were given in combination (MIC for FC dropped from 1.26 to 0.39 mcg/mL and MIC for PCZ dropped from 0.13 to 0.02 mcg/mL, *p* = 0.0001) [18]. The beneficial interaction was also demonstrated by a reduction in the numbers of colony forming units (CFU) of *C. neoformans* isolates. Combination therapy of PCZ and FC at subinhibitory concentrations, eight fold lower than the respective MICs, significantly reduced the counts below those observed with each drug alone (*p* = 0.0001) [18]. The same study investigated the *in vivo* effects of this combination therapy, and found that it was not significantly more effective than each drug alone in terms of survival [18]. However, tissue burden studies showed that combination therapy reduced the counts significantly below that with each drug used alone. It should be noted that this study only utilized one clinical isolate of *C. neoformans*. In conclusion, because of the genetic variations that exist among these isolates, further investigation of combination therapy using multiple strains is needed.

Another study examined the *in vitro* interaction of AMB with triazoles against *Cryptococcus neoformans.* Synergy was observed in 33% of the isolates for PCZ-AMB combination, compared to 7% for FCZ-AMB and ICZ-AMB combinations [19]. Additivism was noted in 53%, 67% and 73% of the isolates for PCZ-AMB, FCZ-AMB and ICZ-AMB interactions, respectively [19]. Indifference was observed in 14%, 20% and 26% of the isolates for PCZ-AMB, ICZ-AMB and FCZ-AMB combinations, respectively [19]. Antagonism was not observed. This study also investigated the *in vivo* interaction of FCZ-AMB combination and compared it to FCZ and AMB therapy alone. Survival and tissue burden studies revealed that combination therapy was more effective than FCZ therapy (*p* = 0.0001), and equally or more efficacious than AMB therapy alone. *C. neoformans* grown in a medium containing FCZ, with subsequent exposure to AMB, lead to an increase in AMB resistance. However, this antagonism was not observed *in vivo*, and in fact, a positive interaction occurred [19]. This *in vivo* study only used one clinical isolate of *C. neoformans*, and for this reason further studies of this combination treatment with multiple strains should be explored.

## 7. *Candida glabrata*

Terbinafine (TBF) combinations with four triazoles (*i.e.*, FCZ, ICZ, VCZ and PCZ) were examined *in vitro* against weakly susceptible strains of *C. glabrata* to azoles [20]. Synergy was observed in 17% of the TBF-FCZ combinations, 21% of the TBF-ICZ combinations, 33% of the TBF-VCZ combinations, and 12% of the TBF-PCZ combinations [20]. Combinations where synergy was not observed revealed a decrease in the MIC of one or both drugs when given in combinations [20]. Another study evaluated the *in vitro* interaction of PCZ with caspofungin against *C. glabrata* [21]. Synergy was observed in 18% of all the isolates (*n* = 119) including 4% of the isolates resistant to FCZ (*n* = 26) [21]. No antagonism was observed between these drugs. *Candida* resistance has been reported with the older azoles (*i.e.*, FCZ and ICZ) and is now being reported with caspofungin. PCZ and caspofungin may have a role in relapsing *Candida* infections and for FCZ-resistant isolates with high PCZ MICs. Studies are warranted to explore the *in vivo* benefits of this combination therapy.

## 8. *Aspergillus* and *Candida* spp.

The activity of PCZ was tested *in vitro* and *in vivo* against *Aspergillus* and *Candida* strains. Overall, PCZ (MICs ranged from ≤0.002 to 0.5 mcg/mL) was more active than ICZ (MICs ranged from ≤0.008 to 1 mcg/mL) against 39 strains of *Aspergillus* [22]. PCZ was also more active (MICs of PCZ ranged from≤ 0.004 to 16 mcg/mL) than FCZ (MICs ranged from ≤0.062 to >64 mcg/mL) against 275 strains of *Candida* and nine strains of *C. neoformans* [22]. Pfaller *et al.* compared the *in vitro* activities of PCZ, ravuconazole (RCZ) and VCZ to ICZ and AMB against 239 isolates of *Aspergillus* and other filamentous fungi. PCZ was most active, inhibiting 94% of isolates at MIC ≤1 mcg/mL, followed by VCZ (91%), AMB (89%), RCZ (88%) and ICZ (70%) [23]. Another study by Manavathu *et al.* expanded these findings and found that the geometric mean MICs of PCZ against *Aspergillus fumigatus* (0.17 ± 0.11) and *non-Aspergillus fumigatus* (0.16 ± 0.28) were significantly lower (*p* ≤ 0.05) than AMB, ICZ and VCZ. AMB-resistant *Aspergillus* strains were susceptible to PCZ. ICZ- and VCZ-resistant strains showed low level (2 to 3 fold increase in MIC) cross-resistance to PCZ. Time-kill studies against *Aspergillus* demonstrated that the fungicidal activity of PCZ is dependent on time and concentration. This highlights the fact that PCZ is more active than ICZ and VCZ [24].

## 9. *Aspergillus fumigatus*

A study by Manavathu *et al.* evaluated drug combinations of caspofungin with triazoles against *A. fumigatus* [24]. Synergy was observed *in vitro* with PCZ (FICI = 0.32 ± 0.09) and ICZ (FICI = 0.49 ± 0.04) [24]. In contrast, when caspofungin was combined with RCZ (FICI = 0.61 ± 0.31) and VCZ (FICI = 1.61 ± 0.42) there was no interaction [24]. No antagonism was observed. Animal studies are needed to investigate the combination of caspofungin with PCZ or ICZ.

## 10. *Blastomyces dermatitidis*

The activity of PCZ was tested *in vitro* and *in vivo* against *Blastomyces dermatitidis*. PCZ was more active when compared to AMB, ICZ and FCZ [MIC_90_ of 0.06 mcg/mL and minimal fungicidal concentration (MFC_90_) of 4 mcg/mL] [25]. The potency of PCZ treatment was confirmed in a murine model with pulmonary blastomycosis. Survival in mice was prolonged at all PCZ doses (25, 5, or 1 mg/kg) and sterilization of lungs was achieved with AMB (1 mg/kg) and PCZ (only with the 25 mg/kg/day dosing regimen) but not with ICZ (150 mg/kg/day) and FCZ. The potency of PCZ was further exemplified at low doses (1 mg/kg/day) where it was shown to be more effective than ICZ (150 mg/kg/day) in prolonging survival of mice infected with *Blastomyces dermatitidis* [25]. Further studies with human subjects are needed to explore the roles of PCZ in the treatment of *Cryptococcus*, *Aspergillus*, *Candida* and *Blastomyces* infections.

## 11. *Trichosporon* spp.

The activities of AMB, PCZ, ICZ, FCZ, VCZ and RCZ were tested against *Trichosporon* spp. *in vitro* [26]. The fungicidal activities against *Trichosporon* with PCZ, VCZ and RCZ were more active thanAMB and FCZ, but similar to ICZ [26]. Further *in vivo* studies to determine the role of PCZ in the treatment of *Trichosporon* are needed.

## 12. *Scedosporium* spp.

Meletiadis *et al.* compared the *in vitro* activities of the azoles [miconazole (MCZ), ICZ, VCZ and PCZ] to the polyenes (AMB and nystatin) and terbinafine against 13 clinical isolates of *Scedosporium apiospermum* and 55 clinical isolates of *Scedosporium prolificans* [27]. *S. apiospermum* isolates were most susceptible to VCZ (MIC_90_ of 0.5 mcg/mL) followed by MCZ (MIC_90_ of 1 mcg/mL), PCZ (MIC_90_ of 2 mcg/mL) and ICZ (MIC_90_ of 4 mcg/mL). The MIC_90_ values of AMB (16 mcg/mL), nystatin (32 mcg/mL) and terbinafine (>32 mcg/mL) were high. *S. prolificans* isolates were less susceptible (MIC_90_ for all drugs were high (>16) except VCZ, which had a value of 4 mcg/mL). Cross-resistance was observed among all the azoles except PCZ [27]. Another study compared the *in vitro* activities of triazoles (MCZ, VCZ, RCZ, PCZ and UR-9825) to conventional antifungals (AMB, ketoconazole, ICZ and nystatin) against 11 clinical isolates of *S. apiospermum* and 33 clinical isolates of *S*. *prolificans* [28]. The latter group was ineffective against both spp. (MIC_90_ values were >16 mcg/mL), whereas the triazoles showed activity against *S. apiospermum*. RCZ was more active (MIC_90_ of 0.125 mcg/mL) than PCZ or VCZ (MIC_90_ values of 0.25 mcg/mL). Similar to the prior study, *S. prolificans* isolates were less susceptible (MIC_90_ values were 16 for PCZ and RCZ and 4 mcg/mL for VCZ) [28]. Further studies in animal models are needed in an attempt to correlate these important *in vitro* results with *in vivo* data.

## 13. *Coccidioides immitis*

PCZ displayed similar *in vitro* activity to ICZ (MIC range for PCZ was 0.25 to 1 mcg/mL, and for ICZ was 0.125 to 0.5 mcg/mL); however, it was more effective against *Coccidioides immitis in vivo* [29]. Cultures of whole spleens and livers from mice treated with PCZ (10 mg/kg/day) showed ≥70% sterilization, whereas no sterilization was seen with ICZ, even at the higher dose of 30 mg/kg three times daily [29].

## 14. *Mucor* spp.

The *in vivo* activities of PCZ (at doses of 5, 15, and 30 mg/kg twice daily), ICZ (at a dose of 30 mg/kg three times daily), and AMB (at a dose of 1 mg/kg once daily) against *Mucor* spp. were compared in immunocompromised mice [30]. PCZ at doses of 15 and 30 mg/kg twice daily prolonged survival and reduced fungal tissue burden, while ICZ did not. PCZ given at 30 mg/kg twice daily (total daily dose of 60 mg/kg/day) was as potent as AMB [30]. The activity of PCZ is effective in immunocompromised mice; however, further studies are needed to determine its role in the treatment of human coccidioidomycosis and *zygomycosis*.

## 15. *Pseudallescheria boydil*

An *in vitro* study by Gonzalez *et al.* demonstrated that PCZ (MIC_90_ of 1 mcg/mL) was more active against *Pseudallescheria boydil* than ICZ (MIC_90_ of 4 mcg/mL) and FCZ (MIC_90_ of >64 mcg/mL) [31]. Furthermore, there were no statistically significant differences (*p* > 0.05) on survival between treatment with PCZ (at doses of 0.5, 1, 5 and 10 mg/kg once daily) and ICZ (at doses of 30 mg/kg three times daily). However, PCZ was more effective in preventing death (70%–75% survival) at the higher doses (30 or 50 mg/kg once daily and 25 mg/kg twice daily), than FCZ (50% survival at a dose of 20 mg/kg twice daily). This study confirms the relative resistance of PCZ to *Pseudallescheria boydil* [31].

## 16. Dermatophytes

PCZ was considered equally efficacious *in vitro* against dermatophytes when compared to ICZ (MIC_90_ values of 0.5 and 1 mcg/mL, respectively). However, PCZ (MFC_90_ of 1 mcg/mL) showed a higher fungicidal activity than ICZ (MFC_90_ of 2 mcg/mL) against isolates of the *Microsporum* genus (*p* = 0.03). *In vivo* studies are needed to investigate the potential clinical use of PCZ in the treatment of dermatophytes [32].

## 17. Synergy

Studies have investigated the interaction of PCZ with other antifungal regimens against *C. neoformans*, *C. glabrata*, and *A. fumigatus* [18,19,20,21,33]. In these studies, synergy is defined as fractional inhibitory concentration index (FICI) of ≤0.5, additivism as FICI > 0.5 to 1, indifference as FICI > 1 to ≤2, and antagonism as FICI > 2. PCZ has been shown to have synergistic and additivistic activities with other antifungals. These combinations may help avoid the emergence of drug resistance, decrease drug related toxicities with the use of lower doses, expand the spectrum of activity and shorten duration of treatment.

## 18. Postantifungal Effect

Postantifungal effect (PAFE) is defined as fungal growth suppression that persists after exposure to an antifungal drug. It reflects the time it takes for an organism to recover from the exposure to the drug and resume normal growth. This effect varies depending upon the pharmacodynamic interaction between the microorganism and the antifungal agent. In general, fungistatic drugs are expected to produce shorter PAFE compared to fungicidal agents. This is due to the fact that fungistatic drugs, unlike the fungicidal agents, do not cause permanent injury to the fungal cell wall which recovers as soon as the drug is removed.

A comparative study has shown that PCZ produced a short PAFE against *A. fumigatus* (0.75 ± 0.35 h) and *C. albicans* (≤0.5 h) compared to the long PAFE of AMB (7.5 ± 0.7 h and 5.3 ± 1.15 h, respectively) [34]. Although PCZ has fungicidal activity against *Aspergillus*, it has a short PAFE. The fungicidal activity of triazoles is slow (12–24 h for ≥90% killing of cells) due to the prolonged time required for the depletion of lanosterol in the fungal cell by inhibition of CYP450 dependent lanosterol 14α-demethylase. The PAFE is defined on the basis of short exposure (2 h in this study) of the cells to the drug instead of long exposure. The slow fungicidal effect of PCZ explains the short PAFE, in contrast to the rapid fungicidal activity and the long PAFE of AMB [34].

## 19. Pharmacokinetics

### Animal Studies

The pharmacokinetics (PK) of PCZ have been measured in animals (*i.e.*, mice, rats, dogs, monkeys, and rabbits) [35]. After an 18-h overnight fast, these animals were randomly assigned to receive either a single intravenous (IV) or oral (PO) dose of PCZ (20 mg/kg) as a solution or suspension. The IV dose of PCZ was prepared with a 40% aqueous hydroxypropyl-β-cyclodextrin (HPβCD) solution, whereas the PO dose of PCZ was manufactured to either a solution in HPβCD or a suspension containing 0.4% methylcellulose, 0.5% polysorbate 80, and 0.9% sodium chloride (MPS). Blood from research animals were collected according to the study protocol [35]. PCZ concentrations were analyzed by high performance liquid chromatography (HPLC). The results of this study demonstrated that PCZ PO in mice was 100% bioavailable from the HPβCD solution; however, its systemic exposure was 47% from the MPS suspension [35]. The mean area under the concentration time curve (AUC_0–∞_, mcg⋅h/mL**)** values between IV and PO HPβCD solutions were 137 and 143, respectively [35]. Conversely, the mean AUC_0–∞_ for the PO MPS suspension was 64. The bioavailability values of PCZ in MPS **s**uspension and HPβCD solution in rats were approximately 48% and 66%, respectively [35]. Similar results were reported in dogs (37% and 72%, respectively) and monkeys (14% and 52%, respectively). The half-life (t½) values of IV PCZ HPβCD solution were the same at 7 h among mice and rats and were longer among the rabbits (t½ = 9 h), dogs (t½ = 15 h) and the monkeys (t½ = 23 h) [35]. Based on these results, PCZ demonstrated good bioavailability in the animals tested. Results suggest that PCZ PO formulated in the HPβCD solution had a higher gastrointestinal absorption than PCZ formulated in the MPS suspension, suggesting no precipitation had occurred. The authors concluded PCZ may be administered once daily. This dosing is based on the fact that the plasma concentrations of PCZ at 24 h were maintained above the MICs and minimal fungicidal concentrations. However, the above information was not shown in the article.

Various doses of PCZ PO suspensions ranging from 20 to160 mg/kg were given to mice and 10 to 120 mg were administered to rats after feeding [35]. At determined time points, animals (*n* = 3 from each group) were sacrificed and plasma PCZ concentrations were measured by HPLC. Results show a relative dose-dependent effect in the systemic exposure of PCZ PO suspension. This effect was seen at doses of up to 80 mg/kg, in which maximum plasma concentration (C_max_, mcg/mL) and AUC_0–∞_(mcg⋅h/mL) were achieved [35]. Furthermore, as doses increased, C_max_ increases were less than AUC_0–∞_ increases, suggesting that absorption rates reached a plateau at higher doses.

A parallel study conducted by Nomeir and colleagues in fed and unfed dogs demonstrated that food significantly improved the relative systemic absorption (a relative 4-fold increase in C_max_ and AUC_0–∞_) of a single PO dose of PCZ in MPS suspension (at a doses of 10 mg/kg) [35]. A crossover study was subsequently conducted in fed dogs to assess the effect of various PCZ PO doses on plasma concentrations [35]. Each dosing phase (10, 40, 80, and 120 mg/kg) was separated by a 2-week washout period. Similar to the results seen in mice and rats, there was a relative dose related increase in C_max_ and AUC_0–∞_ at doses of up to 80 mg/kg and 120 mg/kg, respectively [35].

Another multiple dosing study was also conducted in fed dogs to investigate the PK of PCZ at 40 mg/kg daily for 8 consecutive days [35]. All blood samples were collected up to 13 days and the plasma PCZ concentrations were measured by HPLC. The authors reported an accumulation index of 2.6 for PCZ PO in MPS suspension. This was based on the mean C_max_ (percent of coefficient of variation, %CV) of 5.6 mcg/mL (43) and 2.1 mcg/mL (31) on days 8 and 1, respectively. However, the mean AUC_0–∞_ values (%CV) remained similar between the two days (107 mcg⋅h/mL (47) *vs.* 105 mcg⋅h/mL (30), respectively) [35]. As a result, the authors suggested that significant accumulation of PCZ was unlikely to occur in multiple daily dosing regimen in fed dogs.

Although the results of these preclinical studies demonstrated the PK of PCZ in animals, it is difficult to extrapolate this data to humans. Furthermore, research on PK, efficacy and safety of PCZ in humans would supplement our knowledge of this agent.

## 20. Dosing Studies of PCZ

The PK of PCZ are summarized in Table 2 and the PK of PCZ from the following studies are displayed in Table 5a and Table 5b. The PK of various PO dosing regimens of PCZ were investigated in two independent, randomized, double-blind, placebo controlled and parallel clinical trials conducted in adult healthy volunteers [36]. The first study was designed in a single dose escalation approach, whereas the latter was conducted in a multiple dosing manner. The study participants (single dose study: *n* = 36, PCZ group and *n* = 18, placebo group; multiple dose study: *n* = 36, PCZ group and *n* = 12, placebo group) remained in the research facility for up to 48 h after the final PCZ dose in the single dose study and the morning dose of PCZ in the multiple dose study. All subjects were followed on an outpatient basis for another 72 h. A series of time specific blood sample collections were performed up to 120 h after PCZ administration in the single dose study, and up to 24 h post-dosing of PCZ on days 1 and 14 in the multiple dosing study according to the research protocol [36]. Plasma PCZ concentrations were measured by HPLC and the PK parameters of PCZ were analyzed by model independent methods. The mean age (range) of both treatment and placebo groups in this study was 23 (18–44) years and the subjects were within 15% of ideal body weight. Except for two females recruited in the multiple dosing study, the rest were males. The race was not specified in both studies.

In the single dose escalation study, PCZ tablets were administered orally in a single dose from 50 mg up to 1200 mg in the morning after a standardised high fat content breakfast. The mean C_max_ (%CV) (ng/mL) of PCZ increased proportionally with the single PO dosing regimen of 50 mg to 800 mg (113 (52) to 1320 (343), respectively). whereas the mean C_max_ of the 1200 mg dose did not show any more increase from the previous levels. Therefore, saturation of PCZ PO absorption was reached at the single 800 mg dose [36]. This observation was also confirmed when comparing PCZ AUC_0–∞_ values (%CV) (ng⋅h/mL) with a single dose of 800 mg *vs.* 1200 mg (49,841 (21,431) *vs.* 45,260 (19,914), respectively) [36]. The mean times to reach PCZ C_max_ values (T_max_ (%CV), h) ranged from 6.3 (3.2) to 6.2 (2.9) with single dose range of 50 to 800 mg, respectively [36]. PCZ had a large mean apparent volume of distribution (V/F (%CV), liters) ranging from 431 (86) to 781 (383) for doses of 100 mg to 400 mg, suggesting significant tissue distribution. However, there was no predictable pattern between tissue distribution and the various doses (Table 5a). Overall, a single dose PCZ tablet is well absorbed systemically but the rate of absorption is relatively slow.

According to these authors, a dose dependent clearance (CL/*F)* was observed with single PCZ doses (50–1200 mg) ranging from 4.1 to 6.6 mL/min/kg [36]. The t½s (%CV, hours) of PCZ ranged from 15.9 (2.9) for the 50 mg to 28.5 (7.4) for the1200 mg doses. These results were similar to those reported in other animal studies [35]. Moreover, the lower doses (*i.e.*, 50- and 100 mg) appeared to have a much shorter t½s than the higher doses (*i.e.*, 200-, 300-, 800- and 1200 mg) [36]. Based on this data, the authors concluded that PO PCZ tablet can be administered in a once or twice daily interval depending on the dosing regimen.

**Table 2:** Mean pharmacokinetics (PK) of Posaconazole (PCZ) after a single oral (PO) dose of 400 mg susp and 400 mg twice daily in healthy volunteers at steady state [2].

	Single Dose	Steady State
Parameter	Value	Value
Maximum concentration (ng/mL)	555	
Average concentration (ng/mL)		723
Time to reach maximum concentration (hours)	5.5 *	5 *
Serum protein binding (%)	98.5	
Volume of distribution (Liters)		3088
AUC_0–X_ (ng⋅h/mL)	11295 ^a^	9093 ^b^
Systemic clearance (L/hr)	43	76.1
Half-life (h)	24.1	31.7
Urinary excretion (%)	14	
Dialyzable	No	
Food or nutritional supplement enhance GI absorption	Yes	

* Data is presented as median; PK = pharmacokinetics; PCZ = posaconazole; PO = oral; susp = suspension; mg = milligram; AUC = area under the concentration time curve from time zero to time X; a = 0 to infinity; b = 0 to 12 h.

The results from the single dose escalation study suggest that frequent administration of PCZ by dividing the dose may lead to improved systemic drug exposure. The authors examined the effects of multiple PCZ dosing in a randomized, placebo controlled study [36]. PCZ was administered at multiple dosing regimens of 50 100, 200, and 400 mg tablets every 12 h for 14 days. The T_max_ after the first and second dose ranged from 5 to 6 and 9 to 11 h and the C_max_ (%CV, ng/mL) ranged from 457 (38) to 4150 (20) and from 371 (30) to 3239 (19), respectively. These results were comparable to those of a single dose regimen (Table 5b) [36]. Steady state of PCZ was reached at day 10 of the study. Accumulation of PCZ was noted with the ratio ranging from 6.6- to 8.3-fold between days 1 and 14 [36].

PCZ appeared to be well tolerated in both single and multiple dosing studies. The most common (≥10%) adverse events reported in the single dose PCZ study included headache, somnolence, and dizziness which were also noted in the placebo group [36]. The frequency of these adverse effects did not increase as the dose of PCZ increased. It should be noted that fatigue (11%) only occurred in the PCZ group. Adverse effects in the multiple dosing study included headache, dry mouth, somnolence, dizziness, fatigue and constipation. Similar findings were reported in the placebo group [36]. Most of the laboratory and diagnostic results, as well as physical examinations, did not change from baseline, with the exception of a few incidents of alterations in liver enzymes. Therefore, the authors concluded that PCZ was safe, well tolerated and could be administered once or twice daily due to its long t½.

## 21. Effects of Empty Stomach

Ezzet *et al*. conducted a randomized, open label, three way crossover study in healthy volunteers (*n* = 18) evaluating the PO bioavailability of PCZ administered at three different dosing regimens [37]. After at least a 12 h overnight fast, subjects who qualified for the study were randomized to receive PCZ suspension either as a single dose of 800 mg (regimen A) or as two doses of 400 mg every 12 h (regimen B), or four doses of 200 mg every 6 h (regimen C). No food was allowed for 24 h after the initial PCZ dose. Instead, 1400 mL IV fluid (5% dextrose in water with 0.45% saline) was administered at 100 mL/hour for caloric and fluid supplementation. Subjects were confined to the research facility for 48 h after the initial PCZ dose until the completion of the last blood sample. Blood samples were collected up to 22, 48, and 36 h after the first dose for regimens A, B and C, respectively, and plasma PCZ concentrations were analyzed by HPLC [37]. Safety and tolerability were monitored throughout the entire research study.

There were eighteen healthy subjects (thirteen black and five white) with a mean age (range) of 36 (26 to 44) years and mean weight (range) of 82 (64 to 100) kilograms [37]. The results of this study showed that regimen C had the highest mean ± standard deviation (SD) C_max_ (405 ± 280 mcg/L), T_max_ (24.2 ± 32 h) and C_min_ (189 ± 135 mcg/L) values measured at 48 h after the initial PCZ dose. Regimen B had the modest mean ± SD C_max_ (225 ± 115 mcg/L), T_max_ (16.6 ± 3.9 h), and C_min_ (96 ± 46 mcg/L) values. Regimen A had the lowest of those values (137 ± 90 mcg/L, 7.8 ± 4.7 h 50 ± 26 mcg/L, respectively) [37]. In this study, the authors were able to characterize PCZ PK parameters in a one compartment PO model using the “goodness of fit approach” [37]. PCZ’s absorption rate constant, absorption t½, elimination rate constant, and terminal elimination t½ were 0.197 h^−1^, 3.5 h, 0.048 h^−1^ and 15 h, respectively [37]. The intersubject coefficients of variation in the absorption rate constants were 18, 60 and 70 for regimens A, B, and C, respectively [37]. Data also demonstrated that the relative bioavailabilities of PCZ 800 mg suspension increased by 98% and 220% with regimens B and C (*i.e.*, 400 mg every 12 h and 200 mg every 6 h), respectively, when PCZ was given under fasting conditions. In addition, minimal adverse effects were reported with all three regimens. The authors concluded that dividing daily administration doses (every 12 and 6 h) significantly increased PCZ exposure under fasting conditions. These results corroborated with the findings from previous studies [36]. Splitting the total 800 mg daily dose of PCZ had better overall absorption as shown by the increased soluble fraction of PCZ and increased gastrointestinal residence time.

## 22. Effects of Food

For optimal absorption, PCZ suspension should be administered with food or a nutritional supplement that contains fat. The effects of a fed state on the relative bioavailability of PCZ was examined in several phase I clinical studies [6,38,39,40]. Courtney *et al.* conducted a randomized, open label, four way crossover PK study in healthy volunteers (*n* = 20) evaluating the effect of food on PCZ PO bioavailability [6]. The dose and formulations of PCZ used in this study were 200 mg/5 mL suspension and 200 mg tablet (two 100 mg tablets). After a 10-h overnight fast, subjects were randomized to receive four different regimens of either a single dose PCZ suspension with or without a high fat (841 calories, 52% fat) or nonfat (461 calories, 0% fat) breakfast or a single dose PCZ tablet with the high fat content breakfast. All meals were consumed within a 20-min period and the assigned treatments were initiated 5 min after. Water was the only fluid allowed as needed during the fasting phase. All subjects were required to complete all four regimens at various time periods and to stay in the research center for 48 h according to the study protocol [6]. Blood samplings were performed at designated periods and the last sample was collected at 72 h post-PCZ administration. Plasma PCZ concentrations and PK of PCZ were analyzed by HPLC and model independent methods, respectively. Safety and tolerance were also monitored throughout the study.

All subjects were 22 to 45 year old, healthy male Caucasians who resided in Germany. The results of this study demonstrated that the mean (%CV) C_max_ (ng/mL) were significantly higher when PCZ suspensions were administered with a high-fat meal than when administered in a fasting state (*p* < 0.001) or PCZ tablets with a high fat meal (*p* < 0.004) [6]. These values in descending order were 512 (34) > 413 (33) > 132 (50), respectively [6]. The mean (%CV) C_max_ of PCZ suspension administered with a nonfat meal was 378 (43), which was also significantly higher than the same formulation administered under a fasting state (*p* < 0.001) [6]. The mean (%CV) AUC_0–72 h_ values (ng⋅h/mL) of PCZ suspension varied among the three controlled conditions as follows: High fat meal *vs.* nonfat meal *vs.* fasted state (13,885 (41) *vs.* 9511 (38) *vs.* 3553 (36)) respectively [6]. Clearly, PCZ is absorbed three to five times greater when administered with meals. The mean AUC_0–72 h_ (%CV) value (ng⋅h/mL) of PCZ tablets when administered with a fatty meal was 10,304 (41), which was significantly lower than the PCZ suspension given with a high fat meal (*p* = 0.001) [6]. Finally, times to reach maximum PCZ concentration (T_max_, h) and t½s (h) were similar for both suspension and tablet formulations ranging from 4.1 (21) to 5.5 (32) and 21 (15) to 23.5 (25), respectively [6].

Headache and fatigue were the two most reported adverse effects with PCZ [6]. This study is limited because subjects were all male and healthy Caucasians less than 45 years. It may be difficult to extrapolate these data to the female gender, other age groups, such as children and elderly, and other races including African Americans and Hispanics. The findings from this study may not be applied to patients with other disease states (*i.e.*, obesity, diabetes mellitus, and HIV/AIDS infection) and patients on medications that alter gastric acid secretion (*i.e.*, antacids, H2 receptor antagonists and proton pump inhibitors). In these situations, multiple dosing PK of PCZ is needed. Moreover, clearance and volume of distribution of PCZ were not determined. The authors concluded that for optimal absorption, PCZ should be administered with food based on bioavailabilities of 168% (2.6 fold) *vs.* 290% (4 fold) with nonfat and high-fat meals, respectively.

The PK parameters of PCZ in the fasted state and after a high-fat meal were also studied by Xu *et al*. in an open-label, randomized, 2 × 2 crossover study that had a washout period of 14 days [38]. A LC-MS method, which was precipitated with 0.6 mL of acetonitrile, was developed in order to measure the PCZ concentrations in human plasma. The lower limit of detection was set to 2 ng/mL. A total of 12 participants were enrolled and were administered 5 mL of PCZ suspension (200 mg) in the fasted state and with a high-fat meal [38]. The study subjects were given a light snack more than 10 h before each treatment on day-1. In the fasting period, food intake was withheld 4 h after the administration of the drug. In the fed phase, a standardised high-fat breakfast was served half an hour before administration of PCZ. Blood samples were drawn pre-administration and at 30 min intervals up to 120 h post-dose [38].

The C_max_ of PCZ ranged between 62.46 and 528.39 ng/mL [38]. In the fasted state, the mean C_max_ was 128.02 ± 92.21 ng/mL compared to 354.29 ± 82.02 ng/mL in the fed state (*p* < 0.05). The corresponding T_max_ levels were 3.88 ± 1.11 and 7.38 ± 2.87 h, respectively (*p* < 0.05). Mean AUC_0–t_ was reported to be 3075.27 ± 1917.00 ng⋅h/mL and 12229.08 ± 2923.37 ng⋅h/mL in the in the fasted and fed states, respectively (*p* < 0.05). The half-life was 22.11 ± 3.02 h in the fasted state compared to 21.39 ± 5.34 h in the fed state. The results demonstrated that the ingestion of a high-fat meal increases the absorption and bioavailability of PCZ without affecting its elimination [38]. This suggests that simultaneous administration of food can lead to higher plasma concentrations of PCZ and, potentially, more effective antifungal activity. This study was limited in that it only included Chinese subjects, which limits the extrapolation of the results to a broader population of patients. Additionally, compliance and the use of concomitant medications were not addressed. It would also be important to determine how the high plasma concentration levels of PCZ translate into better efficacy of the antifungal in future clinical studies.

The effects of food on the PK of PCZ were further evaluated by Lin *et al*. in a phase I, randomized, 3-way crossover study [39]. The objective of the investigation was to analyze the PK parameters of a single dose of 400 mg of PCZ oral suspension given under fasting and fed conditions in healthy male Taiwanese subjects. Study participants (*n* = 16) were administered PCZ 400 mg oral suspension following an overnight fast, or right after a normal-fat meal (700–800 calories; 30% fat), or after a high-fat breakfast (800–1000 calories; 50% fat). There was a washout period of 1 week between each treatment. Blood samples were collected at 0, 1, 2, 3, 4, 4.5, 5, 5.5, 6, 8, 10, 12, 24, 48, 72, 96, and 120 h, and serum plasma concentrations were analyzed using HPLC [39].

A total of 14 participants completed the study and two subjects withdrew after experiencing an adverse event [39]. However, only 13 patients were included in the PK analysis; but the reasons were not stated. The results revealed that C_max,_ AUC _0–t_, and AUC_0–∞_ of PCZ were higher after a normal- or high-fat meal than under fasting conditions. Fasting, normal-fat, and high-fat mean C_max_ values of PCZ were 279.00 ± 123.32 ng/mL, 662.46 ± 251.02 ng/mL, and 608.38 ± 183.22 ng/mL, respectively *(p* < 0.0001); mean AUC_0–t_ values under each condition were 6828.56 ng⋅h/mL, 662.46 ± 251.02 ng⋅h/mL, and 608.38 ± 183.22 ng⋅h/mL, respectively after a normal-fat meal (700–800 calories; 30% fatiepectively (*p* < 0.0001); and mean AUC_0–∞_ values under each condition were 7304.72 ± 3444.54 ng⋅h/mL 21,326.65 ± 8495.01 ng⋅h/mL and 21,626.08 ± 8193.31 ng⋅h/mL respectively (*p* < 0.0001) [39]. Under fasting conditions, the mean T_max_ was much shorter when compared to the T_max_ after a normal or high-fat meal (3.15 h *versus* 4.88 h, respectively; *p* = 0.0178). The mean t½ was 22.0, 20.8, and 22.0 h for each condition, respectively [39]. After analyzing these results, the investigators of the study concluded that the systemic absorption of PCZ increases when administered with food compared to under fasting conditions, and that the parameters are similar when the medication is administered with a normal-fat *versus* a high-fat meal [39]. It is important to note that this study was performed on a small number of Taiwanese patients, limiting the ability to extrapolate the results. Moreover, the clinical relevance of the observed increase in C_max_ and AUC was not discussed.

## 23. Effects of Nutritional Supplements

Sansone-Parsons *et al.* conducted a randomized, open label and crossover study in healthy volunteers (*n* = 23) to evaluate the effects of a nutritional supplement (Boost plus ^TM^) on PCZ exposure [40]. After an overnight fast, subjects were randomized to receive a single dose of PO PCZ (400 mg suspension), either alone or with 240 mL of Boost Plus^TM^ (16% protein, 34% fat and 50% carbohydrate providing 1.5 calorie/mL). After a washout period of at least 14 days, subjects would crossover to receive the alternate treatment. In each study phase, subjects were not allowed to consume any food until 4 h after receiving PCZ and to leave the research center until after the last sample collection at 72-h post dose. A series of time sensitive blood sampling was performed and the plasma PCZ concentrations were measured by LC-MS. PK of PCZ were analyzed by a noncompartmental method. Safety and tolerance of PCZ were monitored during the study period [40].

This study used twenty-four subjects with a mean age (range) of 39.4 (24–53) years and a mean body mass index of 26.2 kg/m^2^ [40]. One of the participants withdrew from the study due to personal reasons. The results of this study demonstrated that co-administration of a nutritional supplement (*i.e.*, Boost plus^TM^) significantly enhanced the relative PO absorption of PCZ by 263% (90% confidence interval: 226–307) than when given alone. The mean (%CV) C_max_ (ng/mL) and AUC_0–72_ (ng⋅h/mL) values were 355 (43) and 9076 (42) *vs.* 121 (75) and 3552 (53), respectively [40]. The T_max_ and t½ values were no different between the two treatment phases. The mean (%CV) total body clearance (CL/F, liters/h) and apparent volume of distribution (V/F, liters) values were lower when PCZ was administered with Boost plus^TM^ (42.6 (56) *vs.* 91.2 (40) and 1573 (51) *vs.* 3674 (51), respectively) [40]. These observations were similar to the data published in previous studies [6,36,37,38,39]. No adverse events were reported. The authors concluded that PCZ taken with any type of food shows enhanced bioavailability (a 2.6 fold increase). As compared to previous trials, there were more Hispanics (16 out of 23) and female subjects (*n* = 12) recruited in this study [6,36,37,38,39].

## 24. Effects of pH and Comedication

Abnormal pH levels have been associated with changes in PK, leading to unpredictable bioavailability and subtherapeutic concentrations of certain medications. Walravens *et al*. performed a crossover study on five healthy subjects in order to determine the impact of pH on the absorption of PCZ and investigate the mechanisms of increased intestinal absorption when an acidic carbonated beverage is coadministered [41]. Study subjects received a single dose of 10 mL of PCZ suspension (40 mg/mL) in four different conditions: With 330 mL of water, with 330 mL of a cola beverage (Coca-Cola), with 330 mL of water after the administration of esomeprazole 40 mg once a day for 3 days, or with 330 mL of Coca-Cola after the administration of esomeprazole 40 mg once a day for 3 days [41]. Blood and gastrointestinal samples were taken at different intervals during the study in order to measure PCZ concentrations and investigate the gastric and intestinal behavior of the antifungal [41].

After analyzing the data, it was shown that administering PCZ with Coca-Cola did not affect the pH of the intraluminal environment when compared to water; however, it did lead to an increase in PCZ gastric concentrations by 102% (*p* = 0.001) and systemic exposure by 70% (*p* < 0.05) [41]. The investigators attributed this effect to a probable increase in PCZ solubility in Coca-Cola and a prolonged gastric residence. Concomitant use of esomeprazole was shown to increase the gastric pH and lead to decreased absorption of PCZ with a drop of 37% and 84% in the mean plasma and gastric AUC values, respectively. Administration of Coca-Cola was not sufficient to compensate for the increase in gastric pH induced by the esomeprazole, since the mean plasma and gastric AUC values were still decreased by 19% and 73%, respectively, when the study participants were given PCZ with both esomeprazole and Coca-Cola. There was a strong correlation between the gastric and plasma concentrations of PCZ (*r* = 0.8165; *p* < 0.0001) showing that dissolution in the stomach is important for the absorption of PCZ [41]. Based on these results, the authors concluded that coadministration of Coca-Cola could serve to increase the bioavailability of PCZ in the fasted state. Nevertheless, they labeled it as only a “partially efficient strategy” for patients that have inadequate food intake and abnormal pH levels due to concomitant use of acid-suppressive agents [41]. Further studies would serve to confirm the impact of pH on the serum concentrations and efficacy of PCZ. However, it is important to note that the PCZ package insert currently recommends the oral administration of PCZ during, or within 20 min, of a meal, with a liquid or nutritional supplement, or with an acidic carbonated beverage [2].

## 25. Protein Binding

PCZ is greater than 98% protein bound to serum albumin [2,36,42]. Protein binding of PCZ was not affected in patients with various stages of chronic kidney disease or in patients with conventional hemodialysis [42]. These values were consistently greater than 98% [42].

## 26. Disposition Studies

Krieter *et al.* conducted a small open label PK study in healthy volunteers (*n* = 8) to examine the absorption, metabolism and excretion of radiolabeled [^14^C] PCZ [43]. Healthy subjects with a mean age of 28 years underwent an overnight fast and received a single dose of radiolabeled [^14^C] PCZ (400 mg suspension, 82 mcCi). PCZ was administered 5 min after the consumption of a standardised high fat breakfast. All subjects remained at the research site for blood, urine and stool sampling and were released after 90% of the administered radioactive dose was excreted and recovered. Recovery times after [^14^C] PCZ administration ranged from 168 h to 14 days post PCZ dose. PCZ was quantified and its metabolites in blood and urine samples were measured by HPLC and LC-MS. The amount of radioactivity from the [^14^C] was assessed by liquid scintillation technique. The PK of PCZ were characterized by model independent methods. Safety and tolerance were monitored during the study period [43].

The PK of PCZ reported in this study were comparable to previous studies [36,41]. The mean (%CV) C_max_ (ng/mL), t½ (hours), CL/F (Liters/hours), V/F (Liters) and CL_R_ (mL/min) were 654 (19), 20 (19), 16.3 (30), 465 (31) and 0.0114 (76), respectively [42]. The median (range) T_max_ (hours) was 9.25, whereas the mean (%CV) AUC_0–∞_ (ng • h/mL) was 26100 (26) [43].

The pooled amounts (%) of radioactivity expressed as mean (%CV) in feces and urine were 76.9 (12) and 14 (11), respectively [43]. After 24 h, the pooled amounts of PCZ and its glucuronide metabolites (M8, M5, and M2) in plasma were 65.7%, 17.5%, 8.97% and 7.88%, respectively [43]. M5 and M2 were diglucuronide conjugates of PCZ, whereas M8 was monoglucuronide. All metabolites of PCZ were inactive. Urinary metabolites (*i.e.*, M8, M9, a monoglucuronide, and M5) accounted for no more than 2% of the administered dose of PCZ. Fecal metabolites contributed up to 94% of the pooled amount of radioactivity which translated into 66.3% of the administered dose. These data suggested that PCZ had minimal interaction with the cytochrome P450 system. Furthermore, PCZ’s metabolism is mediated predominantly through phase II UDP-glucuronosyltransferase (UGT) enzyme pathway, especially UGT1A4 [44].

## 27. P-glycoprotein

The manufacturer database categorizes PCZ as a substrate and inhibitor of P-glycoprotein (P-gp). Sansone Parsons *et al.* conducted a pharmacogenetic study to evaluate the effect of MDR1 gene and its product, P-gp, on systemic exposure of PCZ between black and white subjects [45]. The procedures for the MDR1 single nucleotide polymorphism (SNP) and MDR1 mRNA expression were described in detail in the report. The results of this study showed that the distribution of MDR1 genotypes among the black and white study subjects was similar to the general population [45]. Furthermore, no direct association was observed between any MDR1 single nucleotide polymorphism and the AUC of PCZ. Thus, P-gp did not have a significant influence on the PK of PCZ. Further studies are required to determine the role of other drug transport proteins on the systemic exposure of PCZ.

## 28. Newer Solid Oral Tablet Formulation

Krishna *et al*. investigated the rising single- and multiple-dose PK of PCZ for a new oral tablet formulation in a phase I, randomized, single-center, placebo- controlled study on healthy subjects [46]. The safety and tolerability of the tablet formulation was also evaluated. The study was conducted from 19 March 2009 to 20 May 2009. A total of 25 participants were included in the study and were randomized into two cohorts via a computer-generated sponsor-provided randomization code. Group 1 was administered PCZ 200 mg (*n* = 9) or a matching placebo (*n* = 3) with the following schedule: A single morning dose on day 1, twice daily on day 6, once a day on day 7–14, and twice a day on days 15–22. Group 2 was given PCZ 400 mg (*n* = 9) or a matching placebo (*n* = 3) with the following schedule: A single morning dose on day 1, twice a day on day 6, once a day on days 7–14, but not the 400 mg twice a day on days 15–22 to avoid exceeding the PCZ exposure range that is considered to be safe. Blood samples were collected for both groups on day 1 at 0 h (pre-dose), and at 2, 3, 4, 5, 6, 8, 12, 24, 48, 72, and 120 h post-dose. On day 6, blood samples were taken at 2, 3, 4, 5, 6, 8, 12, 24, and 48 h post-dose. Pre-dose samples were taken on days 12 and 13 of the study. Additional samples were taken for Group 1 on days 14 and 22, and on day 14 for Group 2. The samples were analyzed using liquid chromatography with tandem mass spectrometric (LC-MS) detection with a calibrated range of 5–5000 ng/mL. Safety was addressed via assessment of vital signs, physical examination, electrocardiogram (ECG), clinical laboratory tests and incidence of adverse events throughout the study [46].

Inclusion criteria included male and female between the ages of 18 to 65 years with a BMI of 19 to 35 kg/m^2^. They were required to have blood chemistry, hematology, and urinalysis laboratory test results within normal limits. Female participants had to be post-menopausal, surgically sterilized, or using an accepted method of contraception, and male participants had to be using an accepted method of contraception, or agree to abstain from sexual intercourse during the study and up to 1 month after. Exclusion criteria included a history of an infectious disease occurring 4 weeks prior to the initiation of the study, a positive hepatitis B, hepatitis C, or HIV test, use of medications with a high potential for drug abuse, history of alcohol or drug abuse in that past two years, among many others [46].

The results of this intention-to-treat analysis included 13 subjects in Group 1 (including a replacement subject) and 12 participants in Group 2. They were aged between 31 to 59 years with a mean of 45.9 years and were white and of Hispanic or Latino ethnicity. The analysis revealed that exposure to PCZ is increased in a dose-related manner after both single and multiple oral dose administration of PCZ tablets (200 and 400 mg); mean C_max_ for Group 1 was 778 ng/mL and 1290 ng/mL for Group 2 after single dose administration; and 1800 ng/mL (200 mg QD; Group 1), 2980 ng/mL (200 mg BID, Group 1), and 2940 ng/mL (400 mg QD, Group 2) after multiple dose PO administration. After single administration, mean t½ values were 25 and 26 h for the 200 and 400 mg doses, respectively; C_max_ were reached at a mean T_max_ of 4 and 5 h for the 200 and 400 mg doses, respectively. After multiple dose administration, the accumulation ratio of PCZ in a period of 8 days was approximately 3 for the 200 and 400 mg dose, and around 5 for the 200 mg BID dose. Safety results demonstrated the oral tablet formulation to be generally safe and well-tolerated. A total of 12 study participants (48%) reported at least one treatment-related adverse event. These included elevated hepatic enzymes, diarrhea, headache, dizziness, dry mouth, and nausea; however, none of these adverse events had any clinical sequelae [46].

The authors concluded that the exposure of PCZ from the new oral tablet formulation increased in a dose-related manner with a steady-state C_avg_ exceeding the dose that has been previously considered to be effective against invasive fungal infections (IFIs). They also stated that PCZ tablet is generally safe and well tolerated in healthy individuals [46]. Nevertheless, it is important to consider that the sample size of this study was very small and differential statistics were not employed. Therefore, no p values were reported for any of the data. Also, the study population only included healthy patients of Hispanic or Latino ethnicity, limiting the ability to extrapolate the results to unhealthy patients of other ethnicities.

## 29. Prophylaxis in Neutropenia

Patients with neutropenia resulting from chemotherapy for hematologic cancers and for myelodysplastic syndrome are at risk for invasive and fatal fungal infections. FCZ and ICZ prophylaxis have been the standard of care in order to reduce the incidence and mortality of these infections. FCZ is effective for preventing *Candida* infections but lacks efficacy against filamentous fungi. ICZ has a wider spectrum of activity than FCZ and includes activity against *Aspergillus*, but its use has been limited by the poor tolerability of the oral solution, the erratic bioavailability of the oral capsule and the greater toxicity.

Cornely *et al.* conducted a randomized, multicenter study comparing the efficacy and safety of PCZ (*n* = 304) with those of FCZ (*n* = 240) or ICZ (*n* = 58) prophylaxis in patients with prolonged neutropenia from chemotherapy for acute myelogenous leukemia or myelodysplastic syndrome [47]. Patients were randomly assigned to receive prophylaxis with PCZ (200 mg PO suspension three times daily), FCZ (400 mg PO suspension daily) or ICZ (200 mg PO solution twice daily). Drug prophylaxis was given with each chemotherapy cycle until recovery from neutropenia and complete remission, occurrence of invasive fungal infection or for up to 12 weeks from randomization, whichever came first. Patients who were unable to tolerate the oral study drug were given IV prophylaxis for 3 days or less per chemotherapy. In the PCZ group, the IV drug was AMB deoxycholate (0.3–0.5 mg/kg of body weight), and in the FCZ or ICZ group, the IV prophylaxis was the same dose of the PO drug [47].

The primary end point was the incidence of proven and probable IFIs during the treatment phase, defined as the period from randomization to 7 days after the last dose of the study drug administered during the last chemotherapy cycle [47]. PCZ (2%) was found to be superior in terms of the primary endpoint as compared to the FCZ or ICZ group (8%). Absolute reduction in the PCZ group was −6% with a 95% CI −9.7 to −2.5%, *p* < 0.001 [47]. The secondary end points included the incidence of invasive aspergillosis, mortality from any cause and time to death related to fungal infections. The PCZ compared to the FCZ or ICZ group had significantly fewer patients with invasive aspergillosis (1% *versus* 7%, *p* < 0.001), displayed lower mortality (16% *versus* 22%, *p* < 0.048) and showed a significant benefit (*p* = 0.01) in the analysis of time to death [47]. Serious adverse events, possibly, or probably, related to treatment were reported in 6% of the PCZ compared to 2% of the FCZ or ICZ group (*p* = 0.01). Serious events included QT prolongation, atrial fibrillation, a decreased ejection fraction, and torsades de pointes [47].

The authors mentioned that most of the probable aspergillosis cases were based on a positive *Aspergillus* galactomannan antigen test and not on positive cultures. If these probable cases were removed from the study, PCZ may not have shown to be superior to FCZ or ICZ [48]. There are concerns of *Aspergillus*-resistant strains and cross-resistance with other azoles developing from the use of a broad spectrum drug such as PCZ. This may also translate to the lack of fungicidal activity of VCZ in the treatment of *Aspergillus* infections. It is unknown as to whether aspergillosis occurred as a result of PCZ resistance or other factors, including suboptimal PCZ levels [49]. Sixty-five patients received ICZ, compared to 240 who received FCZ. In lieu of this fact, there may not have been adequate statistical power in this study to conclude that PCZ was better than ICZ [50]. PCZ prophylaxis should probably be reserved for the high-risk patients with hematologic cancers [49].

## 30. Persistent Febrile Neutropenia and Invasive Fungal Infections

Ullmann *et al.* conducted a computer assisted, randomized and open label clinical trial in patients with febrile neutropenia (*n* = 66) or refractory IFIs (*n* = 32) [51]. This study evaluated the PK, safety and tolerability of various dosing regimens of PCZ PO suspension. Most patients in the refractory invasive fungal infection group had experienced treatment failure with caspofungin and/or AMB. The study regimens included PCZ 200 mg four times daily for nine doses, followed by 400 mg twice daily (Group 1); PCZ 400 mg four times daily for nine doses, followed by 600 mg twice daily (Group 2); and PCZ 800 mg twice daily for five doses, followed by 800 mg daily (Group 3). The PK phase of the study was completed after 10 days of treatment, whereas the clinical phase continued up to 6 months in patients with refractory invasive fungal infection or until neutrophil recovery in patients with febrile neutropenia. PCZ concentrations from blood samples in the PK phase were collected on days 3 and 10. On day 10, blood samples were taken at 0 and up to 12-h post dose for all treatment groups. An additional 24-h post dose was done for groups 1 and 2. All plasma PCZ concentrations were determined by LC-MS and PK of PCZ was characterized by compartmental modeling. The efficacy of PCZ was evaluated by clinical response defined by the study criteria [51].

Sixty-nine patients (70%) were assessed for PK analysis on day 3 following the initial high PCZ doses and sixty-one (62%) were assessed on day 10. The results showed that steady state PCZ plasma concentrations among the three dosing groups were not reached on day 3 due to the mean long half-life of PCZ (35 h). On day 10, plasma PCZ steady state concentrations were reached in all three groups. The mean (%CV) systemic exposure (*i.e.*, AUC_τ_, ng • h/mL; C_max_, ng/mL; C_min_, ng/mL; and C_av_, ng/mL) and log-transformed values (NAUC, ng • h/mL) of PCZ in group 1 were the highest compared to those of Groups 2 and 3 [51]. The exposure of PCZ in bone marrow transplant (BMT) recipients was 52% lower than in patients with no history of BMT (191 *versus* 402, ng/mL, *p* < 0.003). The authors explained that this phenomenon might be due to the small sample of BMT patients (*n* = 12). The mean (%CV) apparent clearance (CL/F, liters/h) values were similar among all three groups (283 (354), 179 (82) and 215 (81), respectively) [51].

The baseline characteristics were similar among all the groups with a co-morbid condition of hematologic malignancy (*n* = 89) or BMT (*n* = 32). The percent of refractory invasive fungal infection responders, defined as all randomized subjects who had complete and partial responses, at the end of PCZ treatment were 50%, 10% and 40% in groups 1 (*n* = 12), 2 (*n* = 10) and 3 (*n* = 10), respectively [51]. The success rates of PCZ treatment in febrile neutropenic patients were similar among all study groups (in all randomized patients at the end of therapy the rates were 61% (*n* = 23), 67% (*n* = 21) and 64% (*n* = 22) and, in efficacy evaluable patients after 7 days of therapy, the rates were 76% (*n* = 17), 82% (*n* = 17) and 85% (*n* = 13) for Groups 1,2, and 3, respectively) [51]. Regardless of the dosing regimens, adverse events were headache, edema and rigors (which accounted for 7%), nausea (4%), diarrhea (4%) and vomiting (6%). Adverse effects involving subcutaneous tissue included rash, pruritus, and fissures among all study groups (*n* = 98). Predictable adverse effect patterns were not observed in all study groups with the exception of nausea frequently reported in Group 1 regimen. The overall mortality rate was 22% for all randomized patients with a total of 47% and 11% deaths in the refractory IFIs and the febrile neutropenia groups, respectively [51].

Based on the results of this study, the authors concluded that PCZ was safe and well tolerated [51]. The recommended dosing regimen for PCZ PO suspension was 400 mg twice daily in persistent febrile neutropenia or refractory invasive fungal infection. A much larger and well controlled clinical trial is needed to confirm the clinical outcome of this study.

Several other clinical trials have explored the use of PCZ for the prevention and treatment of IFIs. In a study by Huang *et al*. the administration of PCZ oral suspension for the treatment of IFIs in patients that were refractory to, or intolerant to the first-line therapy, was investigated [52]. It was a multicenter, open label study that included a total of 63 patients [52]. Treatment with PCZ had a clinical response rate of 64.4%, and 52.9% of the patients showed eradication of the invasive fungal infection [52]. The authors reported that the adverse events that were observed were mild-moderate in severity and had a short duration. Therefore, they claimed that PCZ can be used as an alternative or salvage therapy for the treatment of IFIs [52]. Nevertheless, a comparative study would be necessary in order to determine how these clinical response rates compare to those of other antifungals currently used as salvage therapy for IFIs.

In another study, Shen *et al*. investigated the efficacy of PCZ in the prevention of IFIs in a Chinese population [53]. It was a randomized, multicenter study that was conducted in China on patients with acute myelogenous leukemiaand myelodysplastic syndrome who had persistent chemotherapy-induced neutropenia [53]. Administration of PCZ for prophylactic treatment was performed for a maximum of 12 weeks, or until the neutropenia was resolved with complete remission, or until an IFI occurred. Study participants were randomly assigned to receive PCZ oral suspension (*n* = 129) or FCZ (*n* = 123), but only 117 patients in each cohort were included in the statistical analysis. The primary endpoint of the study was the occurrence of proven, probable or possible IFI during the treatment with PCZ. Secondary endpoints included clinical failure rate, all-cause mortality, and the time to first systemic antifungal treatment [53].

The results of this study reported that 11 of 117 (9.4%) of the patients in the PCZ group *versus* 26 of 117 (22.2%) in the FCZ group had an incidence of proven, probable, or possible IFI (*p* = 0.0114) [53]. Moreover, the clinical failure rate was lower in the PCZ group (37/117 (31.6%; 95% CI; 23.3–40.9)) when compared to the FCZ group (49/117 (41.88%, 95% CI; 32.8–51.4)) (*p* = 0.168). Onset of first systemic antifungal treatment was also later in patients receiving PCZ *versus* FLU (*p* = 0.0139). The most commonly reported adverse event was abnormal liver enzymes which was seen in 11 (8.8%) patients in the PCZ group and 6 (5.0%) patients in the FCZ group (*p* = 0.221). The authors of this investigation concluded that PCZ proved to be efficacious and well tolerated in the prophylaxis of IFIs in high-risk neutropenic Chinese patients [53]. However, the results of this study can only be extrapolated to a Chinese population; a larger study with a broader population of patients is guaranteed in order to confirm the clinical relevance of this study.

A more recent study by Kaya *et al*. aimed to compare the efficacy of PCZ to FCZ when used as prophylactic agents in patients with hematological malignancies and high risk for IFIs) [54]. It was a 2 year, retrospective, observational study [54]. High risk for IFI was defined as a deep neutropenia (<100/mm^3^) lasting more than one week. Its diagnosis was based on blood cultures, pulmonary high resolution computerized tomography (HRCT), serum galactomannan (GM) levels, and histopathological analysis. Positive GM levels were defined as >0.5 in two consecutive readings, or >0.7 in a single reading, and they were analyzed twice a week. Positive pulmonary fungal disease and invasive pulmonary aspergillosis (IPA) were diagnosed based on radiological findings, as well as GM positivity for the IPA. Patients with similar characteristics were administered either FCZ 400 mg/day PO (*n* = 70) or PCZ 3 × 200mg/day PO (*n* = 35); the specific PO formulation employed was not noted. Prophylaxis was continued until patients were no longer at high risk for IFIs. Prophylactic failure was defined as the occurrence of IPA or a documented fungal infection despite the use of antifungal prophylaxis, and/or the initiation of empiric antifungal therapy while the prophylaxis was being administered [54].

A total of 105 patients were included in the retrospective cohort with a mean age of 44.5 years; 64% were male and 36% were female [54]. A fungal infection was reported in 22 out the 70 (31%) patients receiving FCZ and 13 out of the 35 (37%) patients who were administered PCZ (*p* = 0.827). Similar results were seen in terms of incidence of IPA, where 21 out of 70 (31%) subjects in the FCZ group *versus* 9 out of 35 (26%) subjects in the PCZ group reported an event (*p* = 0.705). Initiation of empiric antifungal treatment was needed in 21% and 20% of patients in the FCZ and PCZ groups, respectively (*p* = 0.932). Mortality, which was defined as death due to any reason, was reported in 17 (24%) and 4 (8%) patients in the FCZ and PCZ groups, respectively (*p* = 0.195). Differences in frequency of adverse events were not statistically significant between the FCZ and PCZ groups (*p* = 0.276); the most commonly reported included nausea and vomiting. Based on these results, the authors concluded that incidence of IFIs and crude mortality are similar between FCZ and PCZ since no statistical significance was found [54]. They attributed the higher rates of fungal infection and initiation of empiric antifungal therapy in the PCZ group to poor absorption of the drug from the gastrointestinal tract due to nausea and vomiting. The investigators stated that both FCZ and PCZ can be successfully used for fungal infection prophylaxis in patients at high risk [54]. Nevertheless, it is important to note the small sample size of the study and difference in number of study subjects between the two groups that could have affected the results. Also, compliance, use of concomitant medications, and food intake with PCZ was addressed. The study was also conducted in Turkey, limiting its applicability to patients of other nationalities.

## 31. Prophylaxis in Severe Graft-*versus*-Host Disease

Recipients of hematopoietic stem-cell transplants are at increased risk for IFIs. FCZ prophylaxis has become a standard of care for antifungal prophylaxis in the post-transplant patient. An international, randomized, double-blind, multicenter trial conducted by Ullmann *et al.* compared PCZ with FCZ for prophylaxis against fungal infections in patients who had either acute graft-*versus*-host disease (GVHD), chronic extensive GVHD, or being treated with intensive immunosuppressive therapy [55]. Patients (*n* = 600) were randomized to receive PCZ (*n* = 301) PO suspension 200 mg three times daily plus placebo capsules once daily or FCZ (*n* = 299) 400 mg tablet once daily plus placebo oral suspension for a period of 112 days (treatment period). The primary endpoint was the incidence of proven or probable IFIs during the treatment period. Other end points were the incidence of proven or probable aspergillosis, the time to the occurrence of an invasive fungal infection, the overall mortality and the mortality due to fungal infection. The safety and tolerability of the study drugs were also monitored for a period of 24 weeks [55].

PCZ was found to be as efficacious as FCZ in preventing IFIs (incidence of 5.3% *vs.* 9%, respectively; *p* = 0.07) and superior in preventing proven or probable invasive aspergillosis (2.3% *vs.* 7%, respectively; *p* = 0.006) [55]. There was a delay in the onset of infections in the PCZ compared to the FCZ group (mean day 102 *vs.* mean day 88, respectively; *p* = 0.048). The number of overall deaths was similar between the two groups but mortality from IFIs was significantly lower in the PCZ group (1% *vs.* 4% in the FCZ group, *p* = 0.046) [55]. The frequency of drug discontinuation because of adverse effects (34% in the PCZ and 38% in the FCZ group), and the frequency of treatment-related adverse effects (36% in the PCZ and 38% in the FCZ) and serious adverse effects (13% and 10%, respectively) were similar [55].

This study failed to state that patients were excluded from the study if they were unable to take medications orally, since PCZ is only given by mouth. The low incidence of aspergillosis in the FCZ group may be explained by the unintentional selection of less sick patients. Factors such as compliance, diet (intake of a fatty meal can increase PCZ absorption) and serum concentrations were not reported, yet they can have an important impact on treatment success [55].

Provided invasive fungal disease is detected early, treatment is successful with the VCZ and liposomal AMB. For this reason, PCZ prophylaxis in patients with severe GVHD should be reserved for the medical centers that have a low incidence of invasive aspergillosis and have the facilities to pursue a preemptive approach [55].

Winston *et al*. also evaluated the efficacy, safety, and breakthrough infections that are related to long-term treatment with PCZ for the prevention of IFIs in patients with allogenic stem cell transplantation (SCT) [56]. Data was collected from a total of 106 SCT recipients who were administered PCZ as prophylactic treatment. The study was single-center and conducted from 1 January 2007 to 31 December 31 2008 [56]. Study participants were administered an oral suspension of PCZ 200 mg three times a day with meals, whenever possible, from day 1 after the SCT until day 100. For patients unable to take medications orally, itraconazole 200 mg IV q 24 h was temporarily substituted for the oral PCZ suspension. Voriconazole 6 mg/kg IV q 12 h on day 1, followed by 4 mg/kg IV q 12 h was given in place of itraconazole when it became unavailable in the US. Incidence of breakthrough infections was assessed through cultures of blood and other suspected sites of infections, CT scans of chest and abdomen, and *Aspergillus* galactomannan assays. Susceptibility of fungal isolates to PCZ was measured via a microbroth assay, and serum plasma concentrations of PCZ were analyzed by HPLC. Patients were taking several others concomitant medications for their health conditions including chemotherapy, cyclosporine, corticosteroids, methotrexate, mycophenolate, trimethoprim-sulfamethoxazole, atovaquone, maribavir, ganciclovir, and pantoprazole [56].

Results were reported for the 106 patients that were treated and evaluated. Their median age was 46 years and they had many of the factors associated with high risk for IFIs including hematologic disease (56%), unrelated donor (42%), high-dose corticosteroid use (76%), and acute GVHD (60%) [56]. A large percentage of these patients (85%) had oral mucositis during their neutropenic state after the transplantation procedure and before the engraftment, and most were taking pantoprazole during the study (>90%). Study subjects experienced neutropenia for a mean duration of 19.6 days, and prophylaxis with PCZ had a mean duration of 113 days with a range of 1–180 days. A total of 34 patients (32%) had to be changed temporarily to itraconazole or voriconazole during the course of the study due to severe oral mucositis or inability to take medications orally for a mean duration of 9.6 days. Eight of 106 patients (7.5%) had a breakthrough infection during the first 6 months after transplantation, and three other participants had an IFI within 53–66 days after finishing preventive treatment with PCZ; however, these patients were taking another systemic antifungal agent when the infection developed. The onset time for the 11 IFIs was 65 days after the transplantation procedure with a range of 2–162 days. The factors that were associated with the development of these infections included neutropenia (four cases), an infected central IV line (three cases), poor compliance (two cases), administration of PCZ via gastric tube (one case), and engraftment failure (one case). It is important to note that all patients who had an IFI were also taking pantoprazole, and eight of these patients had concurrent diarrhea or mucositis which could have reduced the serum concentrations of PCZ. The most common organisms causing the fungal infections were *Candida* species which was responsible for eight cases, *Aspergillus* species which affected two patients, and a combination of *Aspergillus* species with *Coccidioides immitis* in one patient. Out of the nine infecting isolates that were tested for susceptibility to PCZ, only two showed to be resistant with an MIC >1.0 μg/mL; both were *Candida glabrata*. PCZ plasma concentrations were not initially monitored since a target concentration of PCZ has not been established and a dose of 200 mg three times a day has been proven to be efficacious in previous studies; however, after the development of IFIs in several patients, peak and trough plasma levels of PCZ were measured on days 2, 8, and 15 after transplantation in 15, 14, and 10 patients with neutropenia, respectively. The plasma PCZ concentrations of these patients who developed IFIs were similar to those who did not experience an infection. The reported values of mean peak and trough concentrations were 168 ng/mL and 121 ng/mL on day 2 post-transplantation, 306 ng/mL and 276 ng/mL on day 8, and 257 ng/mL and 211 ng/mL on day 15, respectively. In terms of safety, PCZ was generally well-tolerated with a total of nine patients who experienced nausea and no other adverse events being associated with PCZ administration. After six months post-transplantation, the overall patient-survival was reported to be 65% and fungal-free survival was 62%. There were a total of 37 deaths, with four being associated to the development of an IFI. The most common causes were malignancy relapse, GVHD, and infection [56].

After analyzing these results, the authors concluded that long-term prophylaxis with PCZ after allogenic SCT is safe and associated with few incidences of IFIs [56]. Nevertheless, they expressed their concern with the development of breakthrough infections by PCZ-susceptible organisms even when the recommended prophylactic doses of PCZ were being used. Consequently, the investigators recommended different strategies to increase exposure to PCZ, such as the use of higher doses, administration with an acidic beverage, and limiting the concomitant use of proton pump inhibitors (PPIs) [56]. Future research employing these recommended strategies is warranted in order to determine their impact on the successful prevention of IFIs. Furthermore, it would also be important to determine the statistical and clinical significance of the incidence of IFIs while on PCZ treatment when compared to other antifungals that are currently used for the prevention of these infections.

## 32. Fusariosis

A retrospective analysis of three open label clinical trials included 21 patients with hematological malignancies and other related conditions to evaluate the effectiveness of PCZ in *Fusarium* infections [57]. PCZ was given as an oral suspension of 800 mg per day in 2 to 4 divided doses [57]. All patients had proven or probable invasive fusariosis refractory to (for at least 7 days) or intolerant of (defined as severe infusion-related toxicity, nephrotoxicity, or other organ dysfunction) AMB, which is the conventional antifungal treatment. These patients subsequently received PCZ. Successful response to PCZ therapy occurred in 48% of all patients, and 50% of patients with leukemia [57].

Although there are limitations to a retrospective study, these results show that PCZ is safe, well tolerated and can be used as an alternative to conventional AMB therapy for the treatment of invasive fusariosis [57]. A patient in this study with AMB and natamycin resistant *Fusarium solani* keratitis and endophthalmitis responded well to oral (200 mg PO four times daily) and topical (10 mg/0.1 mL) PCZ treatment. After 3 weeks of therapy, the ocular penetration of PCZ was confirmed by vitreous analysis at a concentration of 0.25 mcg/mL [58]. Another study looked at the *in vivo* effects of PCZ solution at doses of 10 to 100 mg/kg/day against *Fusarium solani* in immunocompetent mice [59]. The results show that treatment with higher doses (50 or 100 mg/kg of body weight/day) were not statistically different from those produced with AMB solution (1 mg/kg of body weight/day) in terms of survival and in reducing the numbers of CFU per gram of kidney and liver [59].

## 33. Oropharyngeal Candidiasis

A multi-center, randomized, investigator-blinded, active treatment study was conducted to compare the efficacy and safety of PCZ and FCZ in HIV/AIDS patients (*n* = 350) who presented with documented oropharyngeal candidiasis [60]. The mean ± SD CD4 cell counts (cells/mm^3^) in both groups were 137 ± 170.5 and 132 ± 160.7, respectively. About 60%–70% of these HIV/AIDS patients were not receiving any antiretroviral medication and the majority (90.2%–92.3%) of isolated fungal cultures at baseline were *C. albicans*. In this study using a modified intent-to-treat analysis, 200 mg daily of PCZ PO suspension (*n* = 178) was found to have similar clinical success (cured and improved condition) as 200 mg on day 1 followed by 100 mg daily of FCZ PO suspension (*n* = 172) at days 7 and 14 of therapy (97% *vs.* 96.9% and 91.7% *vs.* 92.5%, respectively) [60]. In the modified intent-to-treat analysis, randomized participants received one or more doses of PCZ with cultures positive for *Candida* spp. at baseline. Clinical success was defined as resolution (absence of plaques or ulcers and no or minimal symptoms) or partial resolution of pretreatment signs and symptoms of candidiasis based on a scoring system [60]. Similar results were also observed in the per protocol analysis. Although the clinical relapse (defined as ≤20 CFU/mL of *Candida* spp. on day 14 and >20 CFU/mL on day 42) rate appeared to be lower in the PCZ group (31.5%) than in the FCZ group (38.2%), the difference was not statistically significant (*p* = 0.24) [60].

In this study, the rates of mycological success (defined as a quantitative yeast culture yielding ≤ 20 CFU/mL of *Candida* spp.) on days 7 and 14 were similar for both groups (49.7% *vs.* 45% and 60.4% *vs.* 58.1%, respectively) whereas the same rates on day 42 were significantly higher in the PCZ compared to the FCZ group (40.6% *vs.* 26.4%, respectively; *p* = 0.038) [60]. Furthermore, the rates of mycological eradication (defined as zero CFU/mL) were found to be similar in both groups (35.6% *vs.* 24.3%, respectively) with a trend favoring the PCZ group (*p* = 0.084). The discontinuation during treatment and follow up and adverse event rates were similar between both treatment groups. Overall, PCZ PO suspension 200 mg daily was as efficacious as FCZ PO suspension 200 mg on day 1 followed by 100 mg daily given for a total of 14 days for oropharyngeal candidiasis in HIV/AIDS patients [60]. Some of the limitations included small sample size, high risk immunosuppressed patients, changes in immunosuppression status over time, and no antiretroviral therapy and potential drug-drug interactions.

## 34. Invasive *Aspergillus*

Invasive aspergillosis is an infection that significantly affects patients with hematological disorders. According to guideline recommendations, voriconazole is the first-line therapy, and a change in antifungal agent is required as salvage therapy. Heinz *et al*. investigated if the use of PCZ is suitable for the treatment of invasive aspergillosis after voriconazole has failed [61]. It was a retrospective investigation conducted in four different hospitals in Germany [61]. Study subjects were administered sequential antifungal therapy with PCZ after they had received voriconazole. Response rates were measured at 30 and 60 days after initiating PCZ therapy and safety was addressed via measurement of liver enzymes and cholestasis parameters [61].

After analyzing the data, it was shown that PCZ had a success rate of 72.2%; 15 out of 36 (41.7%) patients had a complete response and 11 patients (30.6%) had a partial response at any point in time [61]. A total of eight patients failed treatment and two were not evaluable. Mean liver enzyme levels increased during voriconazole treatment when compared to PCZ treatment; there was an aspartate aminotransferase increase of 31.9 U/L with voriconazole *versus* a decrease of 19.6 U/L with PCZ; alanine aminotransferase was 32.4 U/L with voriconazole and 19.8 U/L with PCZ; gamma-glutamyl transferase levels were 124.2 U/L when compared to 152.3 U/L) with PCZ treatment; and alkaline phosphatase was 71.5 U/L compared to 40.3 U/L with voriconazole and PCZ, respectively. Moreover, there were no study withdrawals due to an adverse event. Based on these results, study investigators concluded that PCZ is both effective and safe as salvage therapy for invasive aspergillosis in patients that had prior administration of another triazole [61]. Nevertheless, it would be important to know if the success rate of PCZ is significant when compared to the success rate of other antifungals that are currently used as salvage therapy. A future study involving differential statistics and a larger sample size would be necessary to further establish the role of PCZ as salvage therapy for invasive aspergillosis.

## 35. Dosing and Administration

PCZ is available in the U.S. as a PO suspension (40 mg/mL). Table 3 summaries the dosing and administration of PCZ. For the treatment of oropharyngeal candidiasis in HIV/AIDS patients of 18 years of age or older, the recommended dose of PCZ is 200 mg PO on day 1 followed by 100 mg PO daily for 13 days based on clinical data comparing its efficacy and safety to FCZ [60]. Patients with persistent febrile neutropenia or refractory IFIs who have failed treatment with caspofungin and/or AMB, are recommended to receive PCZ PO 200 mg suspension four times daily for nine doses, followed by 400 mg PO twice daily up to 6 months or until recovery of neutropenia [51]. PCZ should be given as 200 mg PO three times daily for prophylactic use in immunocompromised adults, including patients with prolonged neutropenia from chemotherapy for acute myelogenous leukemia or myelodysplastic syndrome, acute or chronic extensive graft-*versus*-host disease (GVHD), and/or patients being treated with intensive immunosuppressive therapy. PCZ therapy is continued until recovery from neutropenia and complete remission, occurrence of invasive fungal infection or for up to 12 weeks (febrile neutropenia) or 112 days (GVHD) [47,55]. Since PCZ is primarily metabolised in the liver and has minimal renal elimination, there is no renal dosing adjustment in chronic kidney disease and end-stage renal disease patients on hemodialysis [42,43]. Currently there is no data on PCZ dosing adjustment in hepatic impaired, obese and bariatric surgery patients. PCZ dose should remain the same regardless of age, gender and race [45]. There is still limited data to support PCZ use in pediatrics [2]. During pregnancy and lactation this drug should not be used [2].

**Table 3:** Dosing and Administration of PCZ [2]

Conditions	Dosing and Administration
Oropharyngeal candidiasis in HIV/AIDS	200 mg on day 1 followed by 100 mg daily
Persistent febrile neutropenia	200 mg QID for 2 days followed by 400 mg BID
Refractory invasive fungal infection	200 mg QID for 2 days followed by 400 mg BID
Prophylaxis for prolong neutropenia from chemotherapy	200 mg TID
Prophylaxis for severe graft-*versus*-host disease	200 mg TID

BID = twice daily; TID = three times daily; QID = four times daily.

## 36. Special Populations—Age and Gender

Sansone-Parsons *et al.* conducted a randomized, placebo controlled, blinded study in healthy volunteers evaluating the effect of gender and age on the PK of PCZ suspension (400 mg PO twice daily) [45]. Subjects were stratified into four groups including males or females of 18 to 45 years, and males or females of 65 years or older. After an overnight fast, subjects were randomly assigned to a PCZ or placebo group (drug: placebo ratio = 3:1). The administration of the study drug or placebo occurred 10 min after a high fat meal. Blood samples were collected from the day before drug intake and up to 12 h after the morning doses on day 1 and up to 96 h on day 8. Plasma concentrations of PCZ were determined using LC-MS and PK of PCZ were characterized by model independent methods.

The majority of races in both groups were Caucasians (89% and 94%, respectively). The mean ± SD ages (years) in the non-obese young male and female groups were 30 ± 7.6 and 29.8 ± 10.5, respectively [45]. Contrastingly, the mean ± SD ages (years) in the non-obese elderly male and female groups were 71.3 ± 5.6 and 71.4 (6.1), respectively. The races of the elderly groups were similar to the young adult groups [46]. Pooled elderly subjects (*n* = 24) had a higher, but not clinically significant, PCZ exposure than pooled young subjects (*n* = 24) at steady state (day 8). The mean (CV) log-transformed C_max_ (ng/mL) and AUC_0–12_ (ng • h/mL) values between the two groups were 3374 (38) *vs.* 2607 (40) and 35,444 (39) *vs.* 26,740 (40), respectively [45]. The median T_max_ values ranged from 4 to 6 h, and the mean (CV) t½ and CL/F values were similar between the two groups. The results of this study did not show a difference in the relative bioavailability of PCZ between genders of the same age groups. Adverse effects such as headache (18%) and abdominal pain (10%) were reported to be PCZ-related. Incidences of mild to moderate elevated hepatic enzymes were similar among the study groups. The authors concluded that dose adjustment for PCZ was not necessary in adults and elderly and in male and female patients. This conclusion was based on interpatient variability that could have influenced the results of this study, significant overlap of AUC_0–24_ parameters, as well as small CV values between young and elderly, and male and female subjects.

The most common PCZ adverse effects reported were headache (18%), musculoskeletal pain (12%), abdominal pain (10%), fever (8%), and pharyngitis (8%) [45]. There was no relationship between the occurrence of adverse effects among PCZ groups and age or gender [45].

## 37. Special Populations – Race

Sansone-Parsons *et al.* conducted an open-label and parallel study in healthy volunteers evaluating the effect of race/ethnicity on the PK parameters of PCZ [45]. Subjects who met the study criteria were divided into black and white race groups. After an overnight fast, patients were randomly assigned to receive PCZ or placebo. The administration of study drug or placebo occurred 10 min after a high fat meal. Blood samples were collected before and up to 12 h after the morning doses on day 1 and up to 96 h. Plasma concentrations of PCZ were determined using LC-MS and PK of PCZ were characterized by model independent methods.

The mean ± SD ages (years) in the black and white groups were 36.7 ± 6.7 and 32.6 ± 8.8, respectively, and both groups had similar mean ± SD weights (76.9 ± 7.4 and 75.6 ± 8.8, respectively) [45]. Pharmacokinetic parameters of PCZ were similar between white and black subjects during the initial dosing (day 1) and at steady state periods (day 3). The relative bioavailability values of PCZ were 84.4% (90% CI: 70% to 101% and 84.2% (90% CI: 70% to 101%), respectively [45]. There were no significant differences in the incidence of treatment-emergent adverse events between white and black subjects (71% *vs*. 80%, respectively). The most common adverse events were dry mouth (38%), headache (30%), dry skin (21%), increased frequency of micturition (20%), and insomnia (13%) [45]. No reports of elevated hepatic enzymes were mentioned in this study. The authors concluded that no dosage adjustment of PCZ was needed in different races.

## 38. Chronic Kidney Disease and Hemodialysis

Courtney *et al.* conducted an open-label and parallel group study to compare the PK, safety and tolerability of PCZ 400 mg PO suspension administered as a single dose in healthy volunteers and renal insufficient subjects after a high fat breakfast [43]. The sample size of this study was twenty four with six subjects assigned to each group. Renal insufficiency subjects were further divided into groups based on their creatinine clearance (CL_Cr_, mL/min/1.73 m^2^) as determined by a 24-h urine collection. Groups 1, 2, 3 and 4 consisted of subjects with normal renal function (CL_Cr_ >80), mild renal failure (CL_Cr_ 50–80), moderate renal failure (CL_Cr_ 20–49) and severe renal failure (CL_Cr_ <20 and/or on hemodialysis), respectively [43]. In addition, subjects, who were on hemodialysis, participated in two phases of PCZ dosing (*i.e.*, interdialytic and intradialytic). Each phase was separated by a washout period of at least 3 weeks. Sampling of blood was collected up to 120 h post PCZ dose for all groups, with the exception of Group 4 (interdialytic phase), 96 and 120 h samples were not performed if hemodialysis occurred before these designated time points. During the intradialytic phase (defined as the period after the administration of PCZ and 6 h prior to the initiation of the hemodialysis session) Group 4 blood samples were collected before the PCZ dose and up to 120 h post dose. Pre- and post- dialyzer blood samples were also collected during the dialysis period (~2.5 h). Total urine output was also collected up to 72 h post dosing. Plasma and urine PCZ concentrations were measured by LC-MS and PK of PCZ were determined by model independent methods [43].

In this study, the mean ± SD age (years) and weight (kg) were 52.5 ± 15.5 and 82.1 ± 14.5, respectively, and about 71% of subjects were male, 54% were white, 37% were black and 8% were classified as others [43]. The results demonstrated that the PK parameters were similar among the subjects with normal renal function when compared to those with renal insufficiency but not on hemodialysis [43]. Patients who were on hemodialysis showed a wide variability in PCZ exposure (AUC_tf_, ng⋅h/mL) compared to subjects with normal to moderate renal function. These mean (%CV) values were 14238 (89) to 20826 (96) *vs.* 18425 (42), 16328 (27) and 18613 (34), respectively [43]. This phenomenon was suggested by drug-drug interactions between PCZ and concurrent medications in patients with severe renal insufficiency. The pre-dialysis and post-dialysis total plasma PCZ concentrations measured by AUC_tf_ were similar, suggesting PCZ was not dialyzable. The PK profiles of PCZ during the interdialytic and intradialytic phases were similar. The protein binding of PCZ was > 98% and was not affected by the various degrees of renal insufficiency, including subjects who were on hemodialysis. Adverse effects included somnolence, diarrhea and elevated liver function tests [43].

## 39. Hepatic Impairment, Obesity and Bariatric Surg ery

The PK parameters, safety and tolerability of PCZ in various degrees of hepatic impairment, obesity and bariatric surgery have not been evaluated [2].

## 40. Compromised Gastrointestinal Function

In a study by Cornely *et al*., different dosing strategies that might lead to higher bioavailability of PCZ in patients with compromised gastrointestinal function were evaluated [62]. A secondary objective of this investigation was to determine whether measuring plasma levels of PCZ at an earlier stage in therapy can help predict steady-state plasma levels so they can be used to guide treatment in order to reach the desired threshold level. It was a phase IV, randomized, open-label, comparative, multicenter study in the United States and Europe with a total of 75 patients. Study participants were administered PCZ 200 mg PO three times a day from days 1 through 8. Thereafter, they were randomized into three groups from days 9 through 15. Group 1 received PCZ 200 mg PO TID; Group 2 received PCZ 400 mg PO BID; and Group 3 received PCZ 400 mg PO TID [62]. PCZ was administered 10 min after finishing a meal or an oral nutritional supplement. The primary PK parameter was mean plasma concentration of PCZ, which was defined as the mean of the 0 h (trough, pre-morning dose) and 5 h (post-morning dose) on days 2, 8, and 15. Blood samples were collected on days 1, 2, 3, 8, and 15 in the morning before the dose and five hours post-dose. The mean concentrations of PCZ were measured using a validated LC-MS. The lower limit of detection was set to 5.00 ng/mL with a calibration range of 5.00 to 5000 ng/mL. The target plasma concentrations were 500 and 700 ng/mL on days 8 and 15, respectively. Plasma concentrations of 250 ng/mL and 350 ng/mL on day 2 were selected as desired levels that could result in the steady-state concentrations of 500 and 700 ng/mL. These target PCZ plasma concentrations were chosen based on previous PK studies of PCZ [62].

Inclusion criteria consisted of patients of any sex, race, and age >18 years receiving chemotherapy for acute myelogenous leukemia [62]. Patients also had to be at high risk of poor enteral absorption based on the effects of cytotoxic chemotherapy, such as nausea, vomiting, mucositis, and diarrhea. They also had to be at high risk of invasive IFIs, which was defined as an absolute neutrophil count <500/mm^3^, but free from other significant diseases other than the acute myelogenous leukemia which was confirmed by normal clinical tests. Additionally, female participants had to have a negative pregnancy test at baseline and within 72 h before the start of the study, and be on an accepted method of contraception. Exclusion criteria included moderate-severe liver dysfunction, pregnancy, breastfeeding, and systemic antifungal therapy for proven or provable IFIs. Patients receiving treatment with omeprazole, cimetidine, vinca alkaloids, sirolimus, efavirenz, and anthracyclines, or any drugs that cause QT prolongation were also excluded from the study [62].

Results were provided for 49 out the 75 patients that were enrolled in the study (36 women and 39 men) [62]. The 75 study participants were mostly white (91%) and with a mean age of 53.5 years. Only 49 were included in the final analysis as it was desired to include only those who had no missing PK data on days 3, 8 and 15 (per-protocol analysis); reasons for study withdrawals and missing data were not stated. After evaluating the data, it was decided to use the plasma concentrations from day 3, rather than day 2, as they were found to better predict the concentrations for day 8. Even though there was a risk of compromised absorption due to the pre-existing conditions of the patients, the resulting PCZ plasma concentrations on days 3 and 8 reached the desired levels of 250 and 500 ng/mL, respectively. The mean plasma concentrations of PCZ on days 2, 3, and 8 were 230 ng/mL, 346 ng/mL, and 637 ng/mL, respectively. On day 15 of the study, the levels for the 200 mg TID, 400 mg BID, and 400 mg TID doses were 660 ng/mL, 930 ng/mL, and 671 ng/mL, respectively. By day 8, 37 out of 49 participants (76%) had reached the target level of 250 ng/mL for all dosing regimens of PCZ. Thirty out of 49 patients (61%) reached the target mean plasma concentration of 250 ng/mL on day 3, and 73% of these 30 patients reached the target of 500 ng/mL on day 8. Interestingly, out of the patients who did not achieve this target on day 8, 75% also failed to reach the target level of 500 ng/mL on day 15 of the investigation, demonstrating that administering higher doses of PCZ did not have a significant benefit. Moreover, food intake and fat intake during the study was not enough to provide a significant benefit on increasing PCZ absorption; therefore, no conclusions could be drawn regarding its impact on these poor absorbers. In terms of safety, a total of 74 patients (99%) experienced an adverse event during the study. The most common treatment-related adverse events were nausea [13/75 (17%)], diarrhea [10/75 (13%)], and rash [8/75 (11%)]. Elevated liver enzymes were also reported in five patients, but only considered to be related to treatment in two of these patients. A total of 15 patients did not complete the study due to an adverse event [62].

After analyzing these results, the authors concluded that even with compromised gastrointestinal absorption, a desired plasma concentration of PCZ can be expected in most patients [62]. However, those who were not able to reach the target concentration on day 3, tended to remain poor absorbers through the remainder of the study. Although only hypothesis-generating, the secondary aim of this study revealed that day 3 concentrations are better predictive of day 8 plasma concentrations of PCZ when compared to day 2 levels [62]. Limitations of this study include the use of descriptive statistics which do not provide the ability to see the statistical significance of the data. Also, compliance was not addressed and could have been a potential confounder in the study. Furthermore, restricting the concomitant use of several medications limits the ability to extrapolate the results to patients that have compromised gastrointestinal absorption, but that also need to be in some of these medications for the treatment of other conditions. Future studies could focus on introducing differential statistics that could provide additional valuable information, as well as allowing the use of concomitant medications to determine their impact on PCZ concentrations on patients with compromised gastrointestinal function.

## 41. Pediatrics

The PK data of PCZ in pediatric patients is limited [2]. Daily administration of PCZ 800 mg daily administered in divided doses represents salvage therapy in pediatric patients (*n* = 12) of 8 to 17 years of age with IFIs. Mean (median, range) steady-state concentrations (ng/mL) with this dose were 776 (580, 85.3–2891). These values were similar to those in adult patients who received the same dosing regimen [817 (626, 0–3710)] [63]. These results show that there is a large intersubject variability within each population. However, the study did not state whether PCZ was administered with or without a meal [63]. Furthermore, the efficacy and safety of the drug against invasive fungal infection were investigated in the same study groups. Despite the small sample size, the overall success rate in the pediatric group (45%, *n* = 11) was similar to the adult group (50%, *n* = 319) [2,63]. Adverse effects of PCZ in the pediatric population were primarily gastrointestinal related, fever and headache, which were comparable to the events in the adult group [2,63]. The authors concluded that PCZ was an effective salvage therapy in children with refractory IFIs. The clinical data on the use of PCZ in pediatric population is still limited [2].

## 42. Mechanism of Resistance

One major cause of resistance to azole antifungals is decreased binding via alteration of the CYP51A gene that encodes the enzyme lanosterol 14α-demethylase. This explains why the susceptibility of *C. albicans* isolates to azole antifungals (*i.e.* FCZ, ketoconazole, and ICZ) has decreased [64]. Another cause of resistance to these drugs is the reduced intracellular azole accumulation due to the expression of a membrane bound efflux pump [64].

Resistance to azole antifungal agents developed *in vitro* when amino acid substitutions occurred at specific positions in the binding site of CYP51A from *A. fumigatus* (AF-CYP51A) and *C. albicans* (CA-CYP51A) [65]. MIC values increased 100 fold with both PCZ and ICZ (MICs ranged from 0.5 to >16 mcg/mL) when Gly54 position was replaced with either Arg, Glu or Trp. This was seen in mutant forms of AF-CYP51A compared to the wild type isolates (MICs ranged from 0.03 to 0.12 mcg/mL) [65]. However, there was no change in the MIC values with VCZ. The long side chains of PCZ and ICZ (the triazole-3-one five membered ring) fit securely within the helix A′, the FG loop and β turn of CYP51A [65]. The replacement of Gly54 with amino acids that have side chains, such as Arg, Glu or Trp, created Van der Waals forces with the side chains of PCZ and ICZ. This resulted in alteration of receptor binding and increased MIC values. A 64 fold increase in MIC (mcg/mL) values was noted with VCZ when Gly138 and Gly448 were located near the heme binding vicinity, or were replaced with Arg and Ser, respectively [65]. An eight-fold increase in MIC (2 mcg/mL) was reported with ICZ when Gly138 was substituted for Arg compared to the wild type isolate (MIC = 0.25 mcg/mL). However, when that same amino acid substitution occurred, it did not alter AF-CYP51A and the MIC values with PCZ.

Two clinically FCZ-resistant strains of *C. albicans* revealed amino acid substitutions in CYP51. The first strain revealed substitutions at Tyr257His and Gly464Ser and the second strain revealed substitutions at Lys128Thr, Typ132His, Asp278Asn, and Gly464Ser [65]. Both strains exhibited increased resistance in ascending order to PCZ, ICZ, VCZ and FCZ. The corresponding MICs (mcg/mL) values ranged from 0.5 to 128 in the first strain and 0.25 to 32 in the second, whereas the resulting MIC (mcg/mL) values for the wild type strains ranged from 0.03 to 0.125. Two additional amino acid substitutions (*i.e.*, Gly30Ser and Ala61Val) in the CA-CYP51 of the first strain resulted in less of an increase in MIC values with PCZ (1 and 4 mcg/mL) and ICZ (4 and 8 mcg/mL) as compared to the values with FCZ and VCZ (both >16 mcg/mL) [65]. Similarly, the amino acid substitution (Pro230Leu) in CA-CYP51 of the second strain displayed a significant increase in MIC values with FCZ and ICZ. The same amino acid substitution lead to an eight fold increase in MIC values with PCZ; however, there was no change in the MIC values with VCZ.

## 43. Safety and Tolerance

Based on the clinical data, PCZ is considered safe and well tolerated. The most commonly reported side effects (>10%) in the small, single and/or multiple dosing PK studies of PCZ, included headache, fatigue, nausea, and dry mouth [36,37,40,42, 43,51,52,57, 66]. Table 4 shows the frequency of any adverse events that occurred in 1% or more of patients treated with either PCZ or FCZ [47,55,60]. These events seen in the larger efficacy and safety clinical trials, were similar between the two antifungals [47,55,60].

**Table 4:** Any adverse effects (≥1% of PCZ) in large, randomized, double-blind, parallel clinical trial evaluating PCZ *versus* fluconazole (FCZ) in Prophylaxis in patients with neutropenia, severe graft-*versus*-host disease and treatment of oropharyngeal candidiasis in patients with HIV/AIDS [47,55,60].

	PCZ	FCZ
Adverse Event	*n* = 783	*n* = 711
Nausea	5.0	6.0
Diarrhea	3.1	4.5
Neutropenia	2.8	2.5
Headache	2.0	3.1
Bilirubinemia	1.9	1.0
Increased hepatic enzymes	1.4	1.1
Vomiting	1.4	1.5
Fever	1.3	2.0
Hypotension	1.3	2.4
Abdominal pain	1.0	1.1
GI hemorrhage	1.0	0.3

## 44. Pregnancy

PCZ is a pregnancy category C drug [2]. In rats, it is excreted in breast milk and skeletal malformations were observed at doses ≥27 mg/kg [2]. These doses produced a 1.4 fold increase in PCZ steady state plasma concentrations as compared to 400 mg twice daily. In rabbits, teratogenic effects (*i.e.*, bone resorptions) were observed at PCZ doses ≥40 to 80 mg/kg. These doses produced a 2.9 to 5.2 fold increase in PCZ plasma concentrations [2]. Clinical trials have not been conducted in women because of the teratogenic effects in laboratory animals. Therefore, PCZ is recommended during pregnancy only if the potential benefits outweigh the risks to the fetus.

## 45. Drug Interactions

### Antacids

Courtney *et al.* conducted a randomized, open label and four way crossover PK study in healthy volunteers (*n* = 12) to evaluate the effect of antacids on PCZ PO bioavailability [67]. Maximum strength Mylanta, containing aluminum hydroxide and magnesium hydroxide, was used. The antacid dose was 20 mL, providing a total of 102 mEq of acid neutralizing capacity. After 10-h of overnight fasting, all subjects were randomized to receive a single 200 mg PCZ tablet and 200 mL of regular water under four different conditions (either alone, immediately after a standardised high fat meal, antacid alone, or antacid and a standardised high fat meal) [67]. The meal contained 52% fat and provided a total of 841 calories. All meals were consumed within a 20-min period and the assigned treatments were initiated 5 min after meals were completed. Each subject was required to complete all four regimens with a 7-day washout period from the end of the study phase. Subjects were not released from the research center until 72 h after the PCZ dose. Plasma PCZ concentrations were measured by HPLC and the PK of PCZ were determined by model-independent methods. Safety and tolerance were monitored throughout the study.

The study population consisted of non-obese males with a mean age of 34 years. The proportion of black to white patients was a 2:1 ratio [67]. The results confirmed that the antacid did not significantly alter the relative bioavailability of PCZ under all the treatment conditions. In the presence of food and antacid, the mean (%CV) C_max_ (ng/mL) and AUC_tf_ (ng⋅h/mL) values were similar to those in the presence of food only [326 (47) *vs.* 366 (28) and 9513 (45) *vs.* 10220 (31), respectively] [67]. The relative PO bioavailability of PCZ was reported to be 88% [90% confidence interval (CI): 71%–110%]. The same effects were observed in the absence of food with or without antacid. The mean (%CV) C_max_ (ng/mL) and AUC_tf_ (ng⋅h/mL) values were 94.6 (36) *vs.* 92.5 (46) and 2930 (29) *vs.* 2718 (41), respectively [66]. The mean (%CV) T_max_ (h) and t½s (h) were similar among all conditions ranging from 8.5 (41) to 9.0 (47) and 18.3 (14) to 20.4 (18), respectively [67].

The mean (%CV) apparent volumes of distribution (liters) of PCZ were similar in the presence of food with or without the antacid [623 (55) *vs.* 526 (34)] [67]. Similar results were observed in the fasting state, but the values were three times higher than those in the non-fasting state. In addition, the mean (%CV) total body clearances (mL/min) of PCZ were three times higher in the fasting state with or without the antacid than in the non-fasting state with or without the antacid [1072 (18) and 1062 (35) *vs.* 345 (40) and 323 926), respectively] [67]. The authors explained that these observations were due to decreased absorption of PCZ under fasting conditions since the elimination rate constants did not change among all four treatment phases.

The authors concluded that, regardless of the presence or absence of food, PCZ PO absorption was independent of pH. However, the effects on PCZ bioavailability from a more potent acid suppressant, either a H2 receptor antagonist or a proton pump inhibitor, remain to be determined. Furthermore, PCZ PO absorption was enhanced (up to four fold) when PCZ was administered with a meal. Although the number of African Americans in this study was double compared to Caucasians (*n* = 8 *vs. n* = 4, respectively), the PK parameters were similar to those from previous published data, despite a moderate intrasubject variability (29% to 45%) [45,67]. It may be difficult to extrapolate this data to females, other age groups (especially children and elderly), races (including Hispanics and Asians), and to patients with other disease states (obesity, diabetes mellitus, HIV) and other factors (H2 receptor antagonist and proton pump inhibitor). Pharmacokinetic parameters of PCZ from multiple dosing studies are needed.

## 46. Proton Pump Inhibitors

Cojutti *et al*. investigated the influence that dose frequency and concomitant use of proton pump inhibitors (PPIs) have on the time to the PCZ target C_min_ of >700 ng/mL [68]. The study was performed on hematological patients who were administered PCZ prophylaxis after induction chemotherapy for acute myeloid leukemia. It consisted of a retrospective, observational study that was from August 2009 to November 2010 [68]. Inclusion criteria included subjects who had a minimum of three subsequent C_min_ levels during the initial 8 days of treatment. Blood samples were taken before the morning dose and analyzed LC-MS; the lower limit of detection was set to 15 ng/mL. The study had a total of 42 patients that were divided into four cohorts. Group 1 (*n* = 10) received PCZ 200 mg every 8 h without a PPI; Group 2 (*n* = 11) received PCZ 200 mg every 8 h with a PPI; Group 3 (*n* = 10) received PCZ 200 mg every 6 h without a PPI; and Group 4 (*n* = 11) received PCZ 200 mg every 6 h with a PPI [68]. The type of oral formulation administered was not stated.

The results of this investigation reported patients to have similar demographic and clinical characteristics with all *p* values > 0.05 [68]. A therapeutic median C_min_ was achieved by day 4 in Group 3 only, which reported a level of 935.5 ng/mL as compared to 567.0 ng/mL, 420.0 ng/mL, and 514.0 ng/mL in Groups 1, 2, and 4, respectively (*p* < 0.01). On day 8, the differences between the groups were more obvious, with a median C_min_ of 1550.0 ng/mL, 884.5 ng/mL, 716.0 ng/mL, and 589.0 ng/mL in Groups 3, 1, 2, and 4, respectively (*p* = 0.006). These results revealed that increasing the dosing frequency of PCZ, while avoiding the concomitant use of PPIs, can be an efficient approach to achieve a target C_min_ in acute myeloid leukemia patients being administered antifungal therapy for the prevention of breakthrough infections [68]. This was observed when analyzing the results in Group 4, where the expected benefit of increasing the dose of PCZ was counteracted by the concomitant use of a PPI and a subtherapeutic C_min_ level was observed. The authors concluded that avoidance of a PPI with a daily regimen of 200 mg q 6 h can achieve a therapeutic concentration of PCZ and serve as an effective strategy [68].

However, this study was limited in that it included a small sample size that restricts the ability to extrapolate the data to a wider population. Additionally, both increasing the dose frequency and coadministration of a PPI was left to the physician’s discretion, which could have led to an increase in variability. Although not statistically significant, more patients in Group 4 had mucositis; this could have also contributed to the lower concentration of PCZ when compared to Group 2 on day 8 of the study. Nevertheless, this investigation served to demonstrate a potential impact of concomitant use of PPIs on PCZ serum concentrations and warrants future research in the area.

## 47. Cytochrome P450 System Cocktails

The effects of PCZ on the activity of several drug metabolizing CYP450 enzymes were investigated [68]. This study used 13 non-obese adult subjects (age range 18 to 45) who were randomly assigned to receive PCZ (200 mg tablet PO daily) or no drug for 10 days [69]. After a washout period of 14 days, subjects crossed over to the alternate study group. The standardised CYP450 cocktail with corresponding surrogate substrates included caffeine (CYP1A2) 200 mg, tobutamide (CYP2C8/9) 500 mg, dextromethorphan (CYP2D6 and total CYP3A4) 30 mg and chlorzoxazone (CYP2E1) 250 mg. This cocktail was given to both study groups 2 h after the administration of PCZ under fasting conditions on day 9. In addition, midazolam (0.05 mg/kg in 100 mL of 0.9% saline), a hepatic CYP3A4 substrate, was given as a 30 min infusion in place of the CYP450 cocktail on day 10 [69]. Blood samples were collected before and up to 24 h, and urine collection started 2 h and up to 48 h post-PCZ administration according to the research protocol [69]. Plasma PCZ concentrations were measured by HPLC and PK of PCZ were described by model independent methods. Concentrations of the CYP450 substrates and their metabolites were measured by HPLC and LC-MS based upon the specific enzyme isoforms [69]. Safety and tolerance were monitored throughout the study.

Steady state of PCZ was reached after 8 days with a reported mean (%CV) C_max_ (ng/mL), T_max_ (hours), AUC_0–24_ (ng⋅h/mL) and CL/F (mL/min) of PCZ of 1073 (31), 6.9 (21), 19,465 (32), and 188 (34), respectively [69]. Based on the information previously discussed, under fasting conditions, the systemic exposure of PCZ 200 mg PO tablets showed an accumulation index of 1.9, which was less than that reported with the PCZ PO suspension [36]. Plasma- or urine ratios of CYP450 substrates and their metabolites were evaluated to determine the effects of PCZ on the CYP450 isoenzymes. Plasma ratios of 1,7-dimethylxanthine to caffeine and 6-hydroxychlorzoxazone to chlorzoxazone were measured in order to assess the activities of CYP1A2 and CYP2E1, respectively. The urinary ratios of combined 4-hydroxytolbutamide + carboxytobutamide and tolbutamide, dextromethorphan and dextrorphan and dextromethorphan and 3-methoxymorphinan were calculated in order to evaluate the effects of CYP2C8/9, CYP2D6 and total CYP3A4, respectively [69]. Moreover, the hepatic activity of CYP3A4 was measured when midazolam was administered with PCZ. Overall, PCZ did not interact with CYP450 isoenzymes 1A2, 2C8/9, 2D6, 2E1 and total CYP3A4 with the exception of hepatic CYP3A4. The inhibitory effect of PCZ on hepatic CYP3A4 was confirmed by a significant increased mean AUC_0–24_ (%CV) (ng⋅h/mL) of midazolam in the presence of PCZ when compared with the absence of PCZ (93.4 (29) *vs.* 51.4 (29), respectively, *p* < 0.01). This leads to a relative 1.8 (90% confidence interval: 1.57 to 2.14) fold increase in midazolam exposure [69]. Based on the results of this study, the authors concluded that unlike other azole antifungal agents, PCZ has less potential for drug interactions due to its sole inhibition of hepatic CYP3A4.

Although this study suggested that PCZ was a CYP3A4 inhibitor, the magnitude of this effect could not be fully observed since the midazolam was administered as a single dose and PCZ was not given at its optimal dose, absorption state (*i.e.*, with food or nutritional supplement) or in the suspension formulation. In addition, the duration of urine collection may not have been long enough to allow for complete bladder emptying. This may have reduced urine metabolite concentrations. Lastly, the counter effect of midazolam on the PK of PCZ was not examined in this study.

## 48. Cyclosporine and Tacrolimus

Previous studies have shown that PCZ undergoes extensive hepatic metabolism to glucuronide metabolites [42]. PCZ is also a hepatic CYP3A4 enzyme inhibitor [2,69]. Sansone-Parsons *et al.* evaluated the effects of PO PCZ on the systemic exposures of cyclosporine (CSA) and tacrolimus (FK506) in two separate, open label, and single center, drug interaction studies [66]. The CSA-PCZ study was conducted in the United States on heart transplant recipients (*n* = 4) who had normal cardiac function; whereas the FK506-PCZ study was performed in England on healthy volunteers (*n* = 36). Subjects were excluded from the studies if they tested positive for hepatitis B surface antigen, hepatitis C antibody and human immunodeficiency virus antibodies. Additional exclusion criteria included subjects with a history of mental illness, vegetarian diet, current smoking, use of alcohol, or other medications. All study drug concentrations were analyzed by LC-MS [66,70].

**Table 5a:** Pharmacokinetics of PCZ in Different Single Dose Clinical Studies.

Ref	Sample Size	Subjects	Study Design	PCZ Dose (mg)	Dosage Form	With Breakfast (HF)	Cmax (ng/mL)	Tmax (h)	AUCtf (ng.h/mL)	AUCX-Y (ng.h/mL)	t½ (h)	CL/F (mL/min)	V/F (Liters)
							Mean (% CV)	Mean (%CV)	Mean (%CV)	Mean (%CV)	Mean (%CV)	Mean (%CV)	Mean (%CV)
[6]	20	Healthy	O, R, C	200	Susp	No	132 (50)	5.0 (149)		3553 (36) ^b^	23.5 (25)		
				200	Susp	Yes (NF)	378 (43)	4.1 (21)		9511 (38) b	22.2 (18)		
				200	Susp	Yes	512 (34)	4.8 (9)		13885 (41) ^b^	23 (19)		
				200	Tab	Yes	413 (33)	5.5 (32)		10304 (41) ^b^	21 (15)		
[36]	54	Healthy	O, PL, P, DB, R	50	Tab	Yes	113 (46)	6.3 (51)	2317 (50)	2501 (45) ^a^	15.9 (18)	389 (40)	511 (32)
				100	Tab	Yes	235 (26)	7.3 (36)	6101 (28)	6357 (27) ^a^	18.3 (13)	275 (21)	431 (20)
				200	Tab	Yes	332 (21)	5.8 (35)	10354 (30)	10896 (31) ^a^	24.5 (22)	341 (40)	674 (18)
				400	Tab	Yes	611 (31)	6.3 (44)	19401 (33)	20264 (33) ^a^	24.1 (24)	363 (35)	781 (49)
				800	Tab	Yes	1320 (26)	6.2 (46)	46984 (40)	49841 (43) ^a^	24.4 (33)	320 (48)	594 (19)
				1200	Tab	Yes	933 (28)	8.8 (85)	41755 (42)	45260 (44) ^a^	28.5 (26)	585 (73)	1341 (58)
[38]	12	Healthy	O, R, C	200	Susp	Yes	354.29 ^f^	7.38 ^f^		12612.35 ^a,f^	21.39 ^g^		
						No	128 ^f^	3.38 ^f^		3196.18 ^a,f^	22.11 ^g^		
[39]	16	Healthy	R,C	400	Susp	Yes	608.38 ^h^	4.88 ^i^		21626.08^a^	22.0		
				400	Susp	No	279.00 ^h^	3.15 ^i^		7304.72^a^	22.0		
[40]	23	Healthy	O, R, C	400	Susp	Yes (Boost)	355 (43)	5 (4–8) *		11295 (40) ^a^	26 (19)	710 (56)	1574 (51)
				400	Susp	No	121 (75)	4 (2–12) *		5258 (48) ^a^	27.3 (26)	1520 (40)	3674 (51)
[43]	8	Healthy	O	400	Susp	Yes	654 (19)	9.25 (26)		26100 (26) ^a^	20 (19)	271.7 (30)	465 (31)
[42]	24	CLCR > 80	O, P	400 mg	Susp	Yes	555 (40	5.5 (5–8) *		18425 (42) ^a^	24.1 (22)	421.7 (42)	834 (34)
		CLCR 50–79		400 mg	Susp	Yes	631 (47)	5 (5–6) *		16328 (27) ^a^	28.1 (22)	436.7 (30)	1073 (37)
		CLCR 20–49		400 mg	Susp	Yes	486 (37)	8 (5–12) *		18613 (34) ^a^	29.6 (17)	406.7 (45)	1034 (49)
		Interdialytic HD period		400 mg	Susp	Yes	809 (93)	5.33 (15)	20826 (96)	14751 (140) ^a^	68 (14)	1415 (81)	2893 (96)
		Intradialytic HD period		400mg	Susp	Yes	548 (84)	5.42 (17)	14238 (89)	14656 (115) ^a^	76 (13)	1638.3 (105)	3061 (99)
[45]	10	Healthy	R, PL	200	Tab	No	778 (29)	4.0 (3–8) ^d^	23000 (23)	23000 (23) ^c^	25.1 (20)	8.80 (26) ^e^	
	9			400	Tab	No	1290 (29)	5.0 (3–8) ^d^	42800 (35)	42800 (35) ^c^	26.1 (22)	9.55 (34) ^e^	

PL = Placebo, O = open label, P = parallel, R = Randomized, C = Crossover, DB = double blind, B = Blind, NF = Non-fat, HF = High-fat, IF = If possible, Tab = Tablet, Susp = Suspension; HD = Hemodialysis, mg = milligrams, ng/mL = nanogram per milliliter, ng⋅h/mL = nanograms x hour per milliliter, mL/min = milliliter per min, %CV = percent coefficient of variation, * Median (range), CLCR = Creatinine clearance (mL/min); Cmax = Maximum concentration, Tmax = Time to reach Cmax; t½ = half-life, CL/F = Apparent total clearance, V/F = Apparent volume of distribution, AUCtf = Area under the concentration time curve from time zero to the time of recovery of the final sample with a quantifiable concentration, AUCx-y = Area under the concentration time curve from time X to a specific time Y, ^a^: X-Y is from 0 to infinity; ^b^: X-Y is from 0 to 72 h; c: X-Y is from 0 to 24 h; ^d^: median (range); ^e^: results reported in L/h; ^f^: *p* < 0.05; ^g^: *p* > 0.05; ^h^: *p* < 0.0001; *p* = 0.0176.

**Table 5b:** Pharmacokinetics of PCZ in different multiple dosing clinical studies.

Ref	Sample Size	Subjects	Study Design	PCZ Dose (mg)	Doseage Form	With Breakfast (HF)	Cmax1 (ng/ mL)	Cmin (ng/ mL)	Cav (ng/ mL)	Tmax1 (h)	AUCX-Y (ng.h/ mL)	t½ (h)	CL/F (mL/min)	V/F (Liters)
							Mean (%CV)	Mean (%CV)	Mean (%CV)	Mean (%CV)	Mean (%CV)	Mean (%CV)	Mean (%CV)	Mean (%CV)
[36]	48	Healthy	O, PL, P, DB, R	50 BID	Tab	**Yes**	457 (38)			5 (12)	8295 (36) ^a^	19.2 (16)	225 (34)	365 (29)
				100 BID	Tab	**Yes**	1141 (37)			6 (40)	21,778 (40) ^a^	24.1 (20)	171.7 (24)	343 (24)
				200 BID	Tab	**Yes**	1753 (27)			4 (12)	31,106 (26) ^a^	23.9 (26)	231.7 (34)	467 (32)
				400 BID	Tab	**Yes**	4150 (20)			5 (12)	73,105 (20) ^a^	31 (46)	191.7 (25)	486 (34)
							**Mean (SD)**	**Mean (SD)**		**Mean (SD)**	**Mean (SD)**			
[37]	18	Healthy	O, R, C	800 QD	Susp	No	137 (90)	50 (26)		7.8 (4.7)	3900 ^a^			
				400 BID	Susp	No	225 (115)	96 (46)		16.6 (3.9)	770 ^a^			
				200 QID	Susp	No	405 (280)	189 (135)		24.2 (3.2)	12,400 ^a^			
[45]	24	Healthy adults	B, R, PL	400 BID	Susp	Yes	2607 (40)			5 (4–8) *	26,740 (40) ^b^	31.6 (18)	551.7 (29)	
	24	Healthy elderly		400 BID	Susp	Yes	3374 (38)			5 (0–12) *	35,444 (39) ^b^	41.8 (27)	445 (45)	
[45]	13	Young male	B, R, PL	400 BID	Susp	Yes	2682 (51)			4 (4–8) *	27,784 (51) ^b^	31.3 (21)	558.3 (22)	
	11	Young female		400 BID	Susp	Yes	2517 (18)			5 (4–8) *	25,507 (20) ^b^	31.9 (14)	543.3 (22)	
	12	Elderly Male		400 BID	Susp	Yes	3250 (36)			5 (0–6) *	32,924 (35) ^b^	37.1 (21)	468.3 (45)	
	12	Elderly female		400 BID	Susp	Yes	3498 (40)			6 (0–12) *	37,924 (43) ^b^	42.4 (NC)	423.3 (46)	
[45]	28	Blacks	O, R, P	400 BID	Susp	Yes	2857 (36)			5 (4–8) *	30,091 (37) ^b^	37.3 (24)	528.3 (50)	
	28	Whites		400 BID	Susp	Yes	2399 (36)			5 (2–12) *	25,107 (36) ^b^	32.2 (24)	616.7 (44)	
							**Mean (%CV)**	**Mean (%CV)**	**Mean (%CV)**	**Mean (%CV)**	**Mean (%CV)**	**Mean (%CV)**	**Mean (%CV)**	**Mean (%CV)**
[46]	24	Healthy	R, PL	200 QD	Tab	No	1800 (31)		1310 (32)	5.0(2–8) ^♦^	31,400 (32) ^a^			
				200 BID	Tab	No	2980 (38)		2550 (38)	4.0 (2–8) ^♦^	30,600 (38) ^a^			
				400 QD	Tab	No	2940 (46)		2360 (54)	5.0 (0–12) ^♦^	56,600 (54) ^a^			
							**Mean (SD)**	**Mean (SD)**		**Mean (SD)**	**Mean (SD)**			
[51]	69	Persistent FN	O, R, P	200 QID	Susp	Yes, IP	539 (82)		447 (84)	2.65 (0–11.8) *	5314 (83) ^b^			
		Refractory IFI		400 QID	Susp	Yes, IP	531 (71)		423 (70)	5.78 (2–12.4) *	5075 (71) ^b^			
				800 BID	Susp	Yes, IP	417 (60)		340 (63)	4.21 (0–11.9) *	4035 (63) ^b^			
[51]	61	Persistent FN	O, R, P	400 BID	Susp	Yes, IP	851 (82)	640 (98)	723 (86)	3 (0–12.5) *	8619 (86) ^c^	11.9 (3)	4716.7 (354)	2447 (421)
		Refractory IFI		600 BID	Susp	Yes, IP	579 (71)	388 (64)	490 (71)	3.83 (0–10) *	5823 (71) ^c^	12 (3)	2983.3 (82)	4984 (919)
				800 QD	Susp	Yes, IP	361 (74)	252 (100)	259 (72)	4.04 (2.42–12.5) *	6199 (71) ^c^	24 (2)	3583.3 (81)	5061 (903)
[66]	12	Healthy	O, C	200 QD	Tab	No	1073 (31)			6.9 (21)	19,465 (32) ^a^		188 (34)	

PL = Placebo, O = open label, P = parallel, R = Randomized, C = Crossover, DB = double blind, B = Blind, HF = High fat, IF = If possible, Tab = Tablet, Susp = Suspension, M = Males, F = Females, FN = febrile neutropenia, IFI = Invasive fungal infection, mg = milligrams, ng/mL = nanogram per milliliter, ng⋅h/mL = nanograms × hour per milliliter, mL/min = milliliter per min, %CV = percent coefficient of variation, QD = Daily, BID = Twice daily, QID = Four times daily, t½ = half-life, SD = Standard deviation, * median (range), CL/F = Apparent total clearance, V/F = Apparent volume of distribution; ^♦^ median (range), Cmax1 = Maximum concentration after first dose, Cmax2 = Maximum concentration after second dose, Tmax1 = Time to reach Cmax1, Tmax2 = Time to reach Cmax2, AUCtf = Area under the concentration time curve from time zero to the time of recovery of the final sample with a quantifiable concentration, AUCx-y = Area under the concentration time curve from time X to a specific time Y, ^a^: X-Y is from zero to 24 h, ^b^: X-Y is from zero to 12 h, ^c^: X-Y = dose normalized at steady state dosing interval.

In the CSA-PCZ study, heart transplant recipients, who received CSA for 6 weeks or longer, were given PO PCZ 200 mg tablet daily for 10 days [66]. PCZ was administered in the evening on day 1 and after a high fat content breakfast between days 2 to 10. The CSA dose was adjusted for each subject depending on corresponding trough (C_0_) concentration. Blood samples of whole blood CSA (2 mL) concentrations were collected before and up to 12 h after CSA administration on days 1 and 10. Plasma PCZ (4 mL) concentrations were collected before PCZ dosing on days 7 and 8, and at 1 and up to 120 h after PCZ dosing on day 10. In addition, CSA C_0_ concentrations were also measured on days 2, 3, 5, 8, 21 and 28 [66,70].

The subjects in the CSA-PCZ study were all Caucasian males with an average age of 54 years. The investigators reported that the steady state plasma PCZ concentrations increased CSA levels. Thus, CSA dose reductions (ranging from 14 to 29%) were necessary in three out of the four study subjects [66,70]. Consequently, the authors recommended a three-fourths reduction of the CSA dose when starting therapy with PCZ. However, this was based on results from only three study subjects [70]. Also, it was difficult to interpret the results of whole blood CSA concentrations in this study because no statistical analysis was performed and the sample size was small. Furthermore, it was unclear how CSA doses were adjusted since CSA C_0_ concentrations were collected but not reported. Lastly, the PK profile of PCZ was similar to that previously reported in healthy adults [36]. Reported adverse events that were determined to be drug-related in this study included diarrhea, nonherpetic cold sores, dysuria, flank pain, hematuria, urinary tract infection, pain, rash and gastritis [66,70].

Sansone-Parsons *et al.* also examined the effects of PCZ on FK506. Healthy adult subjects received a single PO dose of FK506, 0.05 mg/kg on days 1 and 14 after a 10 h fasting period. PCZ was administered as a 400 mg PO suspension twice daily between days 7 to 14 after a high fat breakfast. [69]. This allowed for a 5 day washout period of FK506. Blood samples for whole blood FK506 were collected before dosing and from 1 to 72 h after FK506 administration on days 1 and 14. Blood samples for plasma FK506 concentrations were also collected on days 1 and 12, before PCZ administration and 12 h after the morning dose on days 13 and 14 [66,70].

The study population consisted of non-obese patients who were 50% male and 92% Caucasians with a mean age of 26 years. The PK profile of PCZ was similar to previous studies [36]. The results of this study showed that PCZ significantly increased the systemic exposure of FK506. The means (%CV) C_max_ (ng/mL) and AUC_0–∞_ (ng⋅h/mL) values of FK506 in the absence and presence of PCZ were 24.2 (31) *vs.* 51.7 (18) and 207 (52) *vs.* 875 (27), respectively [66]. The mean (%CV) t½ and CL/F values of FK506 in the presence of PCZ were 35.9 (10) and 4.31 (30), respectively. The corresponding values of FK506 when given alone were 29 (15) and 21.4 (56), respectively [69]. The median (range) T_max_ (h) values were the same in both study phases [1.5 (1–3)]. The most common adverse events reported during co-administration of FK506 and PCZ were mild-moderate paresthesia (56%) and headache (39%) [66,70].

Despite the similarities in the PK properties of PCZ when given concomitantly with CSA or FK506, there was high intersubject variability of plasma PCZ that was reported (74%–141% and 30% in PCZ-CSA and PCZ-FK506 study, respectively). This limits the ability to draw solid conclusions from these results and creates a need for further research to evaluate this variation in PCZ PK profile. Furthermore, more information is needed on the interaction pathways between PCZ and both CSA and FK506, which are believed to be mediated by inhibition of CYP3A4 and P-gp transport.

In another investigation, Vaes *et al*. analyzed the impact of different factors on PCZ concentrations in myeloid leukemia or myelodysplastic syndrome patients that were being treated prophylactically with PCZ [71]. It was a prospective, bicentric study that was carried out between February 2009 and August 2010. Inclusion criteria included patients 16 and older being treated with PCZ for prophylaxis during their chemotherapy treatment [71]. Study participants were administered an oral suspension of PCZ 200 mg three times a day at the time of meals which were 50 g of fat when possible. The prophylaxis was continued until the neutropenia was resolved (neutrophil count >1000/mm^3^), or until another antifungal medication was initiated for prophylaxis or treatment of IFIs. Blood samples were taken on day 7 of treatment, followed by twice a week thereafter. They were collected before the first dose (time zero [T0].) and 5 h after the first dose (time 5 [T5].) which was correlated with the C_max_. The PCZ concentrations were measured by HPLC. Ongoing treatments with concomitant medications such as proton pump inhibitors (PPIs), histamine (H2)-receptor antagonists, cyclosporine, and tacrolimus were recorded and analyzed. Additionally, the effects of other factors such as age, sex, BMI, comorbidities, and allogenic transplant status were investigated [71].

Results were reported for a total of 40 patients who underwent 74 episodes of treatment with PCZ [71]. Treatment duration had a median of 22 days and ranged from 4 to 93 days. After analyzing the PCZ concentrations for 20 of the study participants, it was confirmed that there was no difference in the results from T0 to T5 (*p* = 0.58); therefore, PCZ concentrations at T0 were used from that point on in the study. Additionally, study subjects were not able to eat more than 30 g of fat in a meal; therefore, a high-fat meal was considered to be one with more than 20 g of fat. PCZ concentrations revealed that age, sex, and BMI do not affect serum plasma levels of the drug (*p* = 0.44, *p* = 0.62, *p* = 0.12, respectively). Allogenic transplant status also did not have an impact on PCZ concentrations when compared to other patients (*p* = 0.33). However, patients with mucositis of grade 1, 2, and 3 had lower PCZ concentrations at 270, 280, and 200 ng/mL, respectively, when compared to patients without mucositis who had PCZ levels with a median of 430 ng/mL (*p* < 0.001). Caloric intake was also shown to affect serum plasma levels of PCZ with study participants who had a caloric intake >1000 kcal/day having a level of 470 ng/mL compared to 300 ng/mL in patients with reduced caloric intake (*p* = 0.02) [71]. In regards to concomitant use of medications, patients taking PPIs had a lower PCZ concentration at 390 ng/mL compared to 510 ng/mL for patients who were not being administered a PPI (*p* = 0.02). Moreover, the concomitant use of ranitidine or cyclosporine did not affect PCZ concentrations (*p* = 0.07 for both). On the contrary, the use of tacrolimus led to an increased PCZ concentration (*p* = 0.03), with patients taking both medications having an average level of 160 ng/mL higher than patients not taking tracolimus (*p* < 0.001). Incidence of adverse events was not addressed [71].

Based on these results, the authors concluded that the main factors that have an impact on PCZ concentrations include the severity of mucositis, caloric intake, and concomitant use of PPIs or tacrolimus [71]. They recommended the use of H2-receptors antagonists instead of PPIs when possible, as well as further research to confirm the effect of tracolimus since the higher PCZ concentrations were observed in only two patients [71]. However, this study had some limitations, including a small sample size and lack of optimization of the PCZ dose which could have allowed for patients to reach higher serum plasma concentrations of the drug. Additionally, the different factors that were analyzed were not labeled as primary or secondary outcomes; therefore, it cannot be determined whether the study was actually powered to find a difference between any of them. Also, baseline characteristics were not presented with *p* values, limiting our ability to determine if the study participants were similar at the beginning of the study. Lastly, there were discrepancies in the amount of measurements that were compared for each of the factors. For example, when determining the effect of concomitant use of tracolimus, only nine PCZ measurements from patients taking the medication were compared to 266 PCZ measurements from patients not on the drug. These limitations warrant the need for further studies in order to define the need for drug monitoring in patients taking PCZ and the extent to which drug interactions with these medications affect PCZ concentrations.

## 49. Rifabutin

Krishna *et al.* performed a nonrandomized, open label, parallel, multiple dose PK study in healthy volunteers (*n* = 24) in order to study the interaction between PCZ and rifabutin at steady state [72]. Subjects were randomly assigned to receive either PCZ alone for 10 days (Group 1) or rifabutin coadministered with PCZ for 10 days (Group 2). Study participants received daily doses of PCZ (tablet, 200 mg) and rifabutin (capsule, 300 mg). Steady state levels of rifabutin were established 7 days (days −7 to 1) prior to the administration of PCZ (between days 1–10) in Group 2. Study drugs were ingested in the morning after the consumption of a high fat content breakfast. Plasma PCZ concentrations were determined by a series of time sensitive blood samples collected on day 10 and up to 120 h post PCZ dose in both groups. Steady state PCZ troughs were also done on days 8–10. Contrastingly, plasma rifabutin concentrations were determined from blood samples collected up to 24 h after administration and up to 120 h on days −1 and 10, respectively. All plasma drug concentrations were measured and analyzed using HPLC. Safety and adverse effects were monitored according to the study protocol [70,72].

Study population consisted of Caucasian males with a mean age (range) of 27 (20 to 40) years and with a mean weight of 73 kilograms [72]. In Group 2, four subjects did not complete the study due to adverse events (*i.e.*, leukopenia in 3 subjects and dizziness, headache and fever in 1). In Group 1, the PK of PCZ alone were comparable to previous studies. The mean (%CV) C_max_ (ng/mL), AUC_τ_(ng⋅h/mL) and median (range) T_max_ (h) of PCZ were 766 (37), 12629 (43) and 5 (5–15), respectively [72]. The PK parameters of PCZ were significantly changed with rifabutin (Group 2). The mean (%CV) of C_max_ (ng/mL), AUC_τ_ (ng⋅h/mL), and median (range) T_max_ (h) were 438 (29), 6389 (38) and 7 (3–10), respectively [72]. According to the study, concomitant use of rifabutin reported a 43% and 49% reduction in C_max_ and AUC_τ_ of PCZ, respectively. This reduction in systemic exposure of PCZ was related to a 2-fold increase in PCZ clearance (CL/F, 307 *vs.* 594 mL/min) and a 42% decrease in PCZ t½ (27.4 *vs.* 15.9 h) [72]. In Group 2, the two consecutive mean rifabutin trough concentrations (ng/mL) on day −1 and day 1 of PCZ coadministration were similar (64.1 *versus* 72.1) [70,72].

It is interesting to note that the PK of rifabutin were changed with coadministration of PCZ. The mean (%CV) of C_max_ (ng/mL) and AUC (ng⋅h/mL) of rifabutin were 438 (23) and 3975 (20) respectively, on day −1 and 569 (20) and 6833 (21), respectively, on day 10. The results demonstrated that co-administration of PCZ with rifabutin resulted in a 31% and 72% increase in C_max_ and AUC_τ_ values, respectively. This increase in exposure of rifabutin was associated with a 42% decrease in total rifabutin clearance when compared to rifabutin administered alone (CL/F, 1300 *versus* 758 mL.min) [72].

The frequency of adverse events, which were mild in nature, was higher in Group 2 than in Group 1. In Group 1, the adverse events that were related to drug use included back pain (25%), cough (17%) and dry skin (17%); whereas headache (75%), back pain (67%), leukopenia (42%), eye abnormality (42%) and abdominal pain (42%) were observed in Group 2 [72]. Leukopenia was the only abnormal laboratory finding in both study groups. The investigators concluded that concomitant use of PCZ and rifabutin should be avoided and should only be given together when the benefits outweigh the risks [72]. Close monitoring for adverse events is necessary if PCZ and rifabutin are used together [72].

Some of the limitations of this study were that only healthy Caucasian male subjects were included, sample size was small, and the parallel study design did not account for intersubject variability [72]. Previous PK studies showed that PCZ had a large intersubject variability in the systemic exposure of PCZ. The fact that this study was not conducted in a cross-over manner would not minimize the intersubject variability issue.

## 50. Phenytoin and Cimetidine

Two individual drug interaction studies published in abstract form evaluated the effects of phenytoin and cimetidine on the PK of PCZ in healthy volunteers [73,74]. Due to the limited information provided in these studies, a summary of the results were discussed. The first study by Courtney *et al*. was a randomized, open label, parallel, and multiple dose study in which healthy subjects (*n* = 36) were randomly assigned to three 10 day treatment arms. Group 1 received PCZ 200 mg tablet PO daily, Group 2 received phenytoin 200 mg PO daily, and Group 3 was given the same doses of PCZ and phenytoin [73]. Blood samples measuring plasma concentrations of PCZ and phenytoin and data analysis were performed following study protocol. On day 10, the mean C_max_ (ng/mL) and AUC_0–24_ (ng⋅h/mL) were decreased by 41% and 50%, respectively, after the co-administration of PCZ (200 mg tablet PO daily) and phenytoin (200 mg PO daily) [73]. This was attributed to the increase in clearance of PCZ by 90% when coadministered with phenytoin. On the other hand, the systemic exposure of phenytoin was increased by 16% with concomitant use of PCZ [73]. Based on these results, the authors recommended avoiding the use of phenytoin in patients who are taking PCZ [73].

The second study was a randomized, open label, two-way crossover, multiple dose drug interaction study in which healthy subjects (*n* = 12) were randomly assigned to 10 days of either PCZ 200 mg tablet PO daily in phase 1 or the same dose of PCZ and cimetidine 400 mg PO twice daily in phase 2 [74]. The two phases were separated by a washout period of 7 days. Blood samples measuring plasma concentrations of PCZ and cimetidine and data analysis were performed. The mean C_max_ and AUC_t_ values of PCZ were both decreased by 39% in phase 2 when compared to phase 1 [74]. The relative systemic exposure of PCZ in the presence of cimetidine was estimated to be 61% [90% CI: 54%–59%]. In both treatment phases, the average T_max_ and t ½ was 7 h and 35 h, respectively. The authors concluded that PCZ is safe and well-tolerated as a multiple-dosing regimen, and that absorption is not affected by changes in gastric pH. Nevertheless, concomitant use should be avoided due to the decrease in PCZ relative bioavailability when the pH is 3 or higher [74].

## 51. Glipizide

Courtney *et al.* conducted an open-label, crossover study in order to analyze the effect of glipizide on the relative bioavailability of PCZ [75]. The study was performed on 12 healthy volunteers. Glipizide was administered at a dose of 10 mg on days 1 and 11, and PCZ was given at a dose of 400 mg daily between days 2 to 11 [75]. The results of the study demonstrated that glipizide had no significant effect on the relative bioavailability of PCZ and *vice versa* [70,75]. Consequently, the co-administration of PCZ and glipizide was determined to be safe, even though a non-clinical significant decrease in blood glucose was seen [70,75].

## 52. Caspofungin and Micafungin

Krishna *et al*. investigated the effects of PCZ on the PK of caspofungin or micafungin in a phase I, randomized, parallel study [76]. A total of 67 healthy subjects were randomized into one of two groups. Group 1 was administered caspofungin 70 mg on day 1, followed by 50 mg on days 2–14 as a 1-h IV infusion. Group 2 received micafungin 150 mg once daily on days 1–7 as a 1-h IV infusion [76]. PCZ 400 mg twice a day was administered as an oral suspension on days 1–14 in Group 1, and on days 1–7 in Group 2. The PK parameters that were assessed included C_max_, steady-state AUC over the dosing interval (AUC(t)), and the time to C_max_ (T_max_). Safety was addressed with clinical laboratory tests, vital signs, electrocardiograms, and recording of incidence of adverse events [76].

The results of this investigation revealed that coadministration of PCZ did not lead to affected PK of caspofungin or micafungin [76]. Caspofungin with concomitant use of PCZ reported log-transformed ratio estimates of C_max_ and AUC(t) that were 90% and 98%, respectively, of those with caspofungin alone on day 14 of the study [76]. Similarly, ratio estimates of micafungin coadministered with PCZ were 104% (C_max_) and 109% (AUC (t)) of those with micafungin alone on day 7. There was no change in the median T_max._ The authors concluded that concomitant use of PCZ with caspofungin or micafungin does not affect the PK of either echinocandin, and that it is mostly well-tolerated [76]. This study was limited in that PCZ concentrations were not reported. Future studies could address the effects that both echinocandins have on the PK of PCZ and determine if it has an impact on its efficacy.

## 53. Simvastatin and Midazolam

In a study by Krishna *et al*., the effect of PCZ on the PK of simvastatin and midazolam was investigated [77]. It was a randomized, fixed-sequence, parallel, single-center study performed on 35 healthy subjects. Study participants were randomized to receive oral PCZ 50, 100, or 200 mg [77]. All participants were administered single doses of the reference drug midazolam 2 mg PO alone on day 9; simvastatin 40 mg PO alone on day 6; PCZ 50, 100, or 200 mg PO once daily on days 1–7; PCZ with midazolam on day 8; PCZ alone on days 9–10; PCZ with simvastatin on day 11; and PCZ alone on days 12–13 [77]. The results of this investigation showed that concomitant use of PCZ (at all doses) with simvastatin leads to increased C_max_ and AUC of simvastatin and simvastatin acid when compared to the use of simvastatin alone [77]. The increase was by 5- to 11-fold and by 5- to 8-fold for simvastatin and simvastatin acid, respectively. When compared to the administration of midazolam alone, PCZ (at all doses) inhibits the CYP3A4 metabolism of midazolam, leading to a 3- to 6-fold increase in AUC. The investigators of this study stated in their conclusions that these results confirm the classification of PCZ as a strong CYP3A4 inhibitor [77]. They recommended that the concomitant use of PCZ with simvastatin, and other statins that are mostly metabolised by CYP3A4, should be avoided, and that other statins that are not affected by CYP3A4 inhibition be used instead [77]. However, this study did not report any p values in order to determine the statistical significance of the results. Also, it would be important to translate these results into the clinical relevance that an increase of this magnitude in the AUC of simvastatin and midazolam would have in terms of efficacy and safety for patients.

## 54. Benzodiazepines

Heinz *et al*. explored the concentration of PCZ at steady-state when given concomitantly with benzodiazepines in a retrospective study [78]. Blood samples were collected from patients with different indications for antifungal therapy in several clinical settings [78]. Similar plasma concentrations of PCZ were reported from patients receiving both PCZ and temazepam compared to patients who were administered PCZ alone. However, a significant reduction in the concentration of PCZ was observed when coadministered with lorazepam compared to PCZ alone with median values of 336 ng/mL *vs*. 585 ng/mL, respectively (*p* = 0.001) [78]. Patients who received a total daily dose of PCZ of 800 mg also showed a similar trend with a concentration of 292 ng/mL with PCZ and lorazepam compared to 537 ng/mL when PCZ was given alone (*p* = 0.003). However, at a lower dose of 200 mg three times a day, the difference in plasma concentrations of PCZ was not statistically significant, even though the same pattern was observed (512 ng/mL *versus* 689 ng/mL; *p* = 0.186) [78]. Based on these results, the authors claimed that concomitant use of lorazepam with PCZ is associated with reduced concentrations of PCZ. They suggested that this could be due to induction of PCZ clearance by glucuronidation [78]. However, the data was not consistent with different doses of PCZ; therefore, further studies would be needed to confirm these results and determine the role of different PCZ strengths and glucuronidation in this potential drug interaction.

## 55. Posaconazole with HIV Antiretroviral Agents

### 55.1. Atazanavir +/− Ritonavir or Efavirenz

In a study by Krishna *et al*., the PK interaction between PCZ and atazanavir, with and without ritonavir, as well as the interaction between PCZ and efavirenz was investigated [79]. It was a phase I, open label, randomized, crossover study that was divided in two parts. In part 1, 12 healthy Caucasian volunteers with an average age of 45 years old were randomized into two groups. Group 1 was administered atazanavir 300 mg and ritonavir 100 mg daily for 7 days, followed by atazanavir/ritonavir with PCZ 400 mg twice a day for 7 days. Afterwards, there was a 28-day washout period after which patients received atazanavir alone daily for 7 days, followed by atazanavir with PCZ daily for 7 days. Patients in Group 2 were administered the same medications in the opposite order. Part 1 of the study was carried out for a total of 59 days [79].

In part 2, 17 healthy Caucasian volunteers with an average age of 43 years old were also randomized into two groups. In Group 1, subjects were administered efavirenz 400 mg daily for 10 days, followed by efavirenz 400 mg daily with PCZ 400 mg twice a day for 10 days [79]. Subsequently, subjects were in a 28-day washout period, which was followed by the administration of PCZ alone 400 mg twice a day for 10 days. In Group 2, patients were administered the same medications in the opposite order. This second part of the study had a duration of 60 days. In both parts of this investigation, PCZ was given after a standardised high fat meal. Assessment of compliance to treatment was not addressed [79].

A one-way analysis of variance model was used to determine the differences in time to reach C_max_ and AUC, which was the primary endpoint of the study [79]. Safety was addressed by incidence of adverse effects and by monitoring of hematology parameters, ECG and vital signs. The power of the study was set to 80% to detect a difference of 22% in AUC in 12 subjects [79].

In part 1 of the study, the results showed that concomitant use of PCZ 400 mg twice a day and atazanavir 300 mg daily for 7 days lead to an increase in mean AUC and mean C_max_ of atazanavir by 3.7 and 2.6-fold, respectively, when compared to atazanavir alone [79]. There was also a delayed systemic absorption by 1.5–2 h, increased half-life (from 5.85 to 11.1 h), and a decreased clearance (from 49.7 L/h to 9.61 L/h) of atazanavir when used in combination with PCZ; this suggested that there is possible CYP3A4 enzyme inhibition by PCZ in the metabolism of atazanavir. Moreover, the addition of ritonavir to both atazanavir and PCZ reported similar results; C_max_ and AUC increased by 1.5 and 2.5-fold, respectively. Also, coadministration of atazanavir, ritonavir and PCZ resulted in 1.5 and 1.8-fold increase in mean C_max_ and AUC of ritonavir, respectively, when compared to atazanavir and ritonavir alone. The investigators attributed these results to a potential UGT1A1 enzyme inhibition by atazanavir in addition to CYP3A4 inhibition by PCZ [79]. Nevertheless, atazanavir and ritonavir did not affect the PK parameters of PCZ. Safety data reported that 92% of the participants experienced ocular icterus, which is possibly related to benign hyperbilirubinemia, an established side effect of atazanavir. An increase in bilirubin that was determined to be clinically significant was reported in a total of 11 subjects [48–156 mmoL/L (baseline bilirubin concentrations were not provided)]. This may have been due to the increase in atazanavir AUC. Other adverse events reported included pollakiuria and abdominal pain (50% and 25%, respectively) [79].

Part 2 of the study showed a 45% and 50% decrease in mean C_max_ and AUC of PCZ, respectively, when coadministered with efavirenz. The authors suggested that this can be due to UGT induction by efavirenz affecting the metabolism of PCZ. On the other hand, PCZ did not have a significant effect on the systemic absorption of efavirenz; it was delayed from 2–4 h when coadministered with PCZ. Commonly reported adverse events included dizziness (47%), fatigue (41%), and headache (41%) [79].

The investigators of this study concluded that there can be a potential increase in plasma concentrations of protease inhibitors and NNRTIs when used concomitantly with PCZ [79]. Their recommendation was to monitor for toxicity and adverse events regularly if coadministration was necessary [79]. Additionally, they did not support the use of efavirenz and PCZ together as their results reported a decreased exposure of PCZ [79]. This study was limited in that it had a small sample size and a short duration. Also, confidence intervals were limited and no p values were provided to show clinical and statistical significance. However, the package insert for posaconazole includes ritonavir, atazanavir, and efavirenz as interacting agents based on the results of this study [2].

### 55.2. Fosamprenavir +/− Ritonavir

Bruggeman *et al.* evaluated the effects of fosamprenavir alone *versus* fosamprenavir boosted with ritonavir on the PK of PCZ and *vice versa* [79]. It was an open-label, three period, cross-over, multiple-dosing study on 24 healthy volunteers with a mean age of 36 years old. The participants received PCZ suspension 200 mg daily on day 1, PCZ 200 mg twice a day on day 2, and PCZ 400 mg twice a day from day 3 forward combined with fosamprenavir 700 mg twice a day or fosamprenavir/ritonavir 700 mg/100 mg twice a day. The treatment regimen was given for a total of 10 days, followed by a washout period of 17 days after which the regimens were switched between the two groups. The power was set to 80% to find a 20% difference in PCZ AUC [79].

The results reported a statistically significant decrease in exposure to PCZ when administered concomitantly with fosamprenavir. The geometric mean ratio (GMR) for PCZ combined with fosamprenavir when compared to administration of PCZ alone was 0.77 (90% CI, 0.68–0.87) and 0.79 (90% CI, 0.71–0.89) for the AUC_0–12_ and C_max_, respectively [79]. No statistical significance was found in the GMR free fraction of PCZ when compared to the combination group (1.10%; *p* = 0.177). The GMR for the AUC_0–12_ and C_max_ were 0.35 (90% CI, 0.23–0.39) and 0.64 (90% CI, 0.55–0.76), respectively, when comparing fosamprenavir with PCZ to fosamprenavir/ritonavir with PCZ [79]. There were a total of 141 adverse events; however, none were reported as serious. Details on these adverse events were not provided. Based on these results, the authors concluded that there is a significant bidirectional PK interaction between PCZ and fosamprenavir. They suggested that this could be due to a reduced exposure of PCZ by fosamprenavir because of UGT1A4 or p-glycoprotein induction, decreased PCZ absorption, or protein displacement of PCZ [79]. Nevertheless, small sample size and short duration of this study limit the ability to determine the long-term effects of concomitant use of PCZ and fosamprenavir. Also, with a study population composed of only healthy volunteers, extrapolating the results to patients with HIV is more challenging. After analyzing these results, it may be concluded that fosamprenavir boosted with ritonavir could be used concomitantly with PCZ; however, therapeutic drug monitoring is recommended. At the present moment, a target plasma concentration of PCZ has not been established due to significant interpatient variability in PK. Consequently, the coadministration should be avoided when possible.

## 56. Posaconazole in HIV-infected Patients

There are a few studies that have analyzed the use of PCZ in the treatment of oropharyngeal/esophageal candidiasis in patients with HIV. Two of these studies investigated the treatment of oropharyngeal candidiasis concentrating on patients who were azole-refractory [80,81]. Exclusion criteria for all trials included patients that had started on a protease inhibitor for the past 30 days. Skiest *et al.* reported that approximately 72% of the participants were receiving antiretroviral therapy [80]. This was a multicenter, international, phase III, open-label study [80]. It was noted that 38% of these patients were taking NNRTIs, 63% were taking NRTIs, and 53% were taking PIs. Study subjects were given a PCZ suspension for 28 days. The primary safety outcome was the incidence of adverse events. QT prolongation was reported as a reason for study withdrawal for at least one patient in the investigation, but the most common adverse events included diarrhea and neutropenia [80]. However, it is important to note that the authors did not discuss how the correlation between study drug use and adverse events was determined, especially with the antiretrovirals. The investigators did mention that the incidence of diarrhea and neutropenia could also be related to the underlying HIV disease, opportunistic infections, or the administration of concomitant medications. Another confounder of this study is related to these concomitant medications that were not listed in detail, even though the percentage of patients taking them was reported. This makes it challenging to determine if there is a direct correlation between the adverse events that were observed to a drug interaction with PCZ.

Another study by Vazquez *et al.* investigated the efficacy of PCZ compared to FCZ for the treatment of oropharyngeal candidiasis [60]. It was a multicenter, randomized, evaluator-blinded clinical study in 350 patients infected with HIV. The participants were administered PCZ or FCZ oral suspension for a total of 14 days. A total of 132 (66%) of these patients were receiving no antiretroviral therapy, 29 (8%) were taking a NNRTI, 117 (32%) were receiving a NRTI, and 85 (24%) were on a PI. Adverse events that were observed and determined to be serious included fever, asthenia, abdominal pain, respiratory insufficiency, and lymphadenopathy [60]. However, the investigators did not consider any of these serious adverse events to be related to the administration of the study drug [60]. As with the first study previously discussed, how the relationship between adverse events and potential drug interactions was determined, particularly with antiretrovirals, was not addressed.

Vazquez *et al.* evaluated the safety and efficacy of PCZ in another study that focused on the long-term treatment of patients infected with HIV and oropharyngeal/esophageal candidiasis who were azole-refractory [81]. The study was a multicenter, international, non-comparative, open-label investigation on a total of 100 patients [81]. Study subjects were administered PCZ oral suspension for a total of 3 months, and the option to continue treatment for up to 12 months for patients who were considered to be clinical responders was offered. At baseline, 70% of the participants were receiving antiretroviral therapy. Out of the patients taking antiretrovirals at baseline, 38% were taking NNRTIs, 64% were on NRTIs, and 48% were receiving PIs [81]. Adverse events reported included neutropenia, urticarial, and cardiac failure/sudden death, and they occurred after 3 months of treatment on a total of three patients. The investigators suggested that these adverse events could have been due to the HIV infection itself, opportunistic infections, or the use of concomitant medications [81]. As with the other two studies, the correlation between study drug use, potential drug interaction, mainly with the antiretrovirals, and the incidence of adverse events was not addressed. The possibility of elevated antiretroviral serum concentrations due to the concomitant use of PCZ remains an area that warrants further research as this increased antiretroviral exposure could have led to the laboratory abnormalities and other adverse events observed in the study.

## 57. Formulary Considerations

Although PCZ has a chemical structure similar to ICZ, it has a wider spectrum of antifungal activity. This drug covers not only selected *Candida* and *Aspergillus* species, but other breakthrough fungal infections including *fusarium*, *scedosporium* and *zygomycosis*. Posaconazole has a unique PK profile that does not require renal dose adjustment. Thus, the drug is considered safe and well tolerated. Posaconazole has been approved by the FDA for the prophylaxis of invasive *Aspergillus* and *Candida* infections in severely immunocompromised patients 13 years and older. However, the efficacy of PCZ in the treatment of IFIs has yet to be determined. The average wholesaler price (AWP) of a 105 mL PCZ oral suspension (40 mg/mL) bottle is $576.00 and the unit cost is $5.49 per 40 mg/mL [82]. Depending on the usage, the AWP cost of a week therapy of PCZ can range from $110 to $770.

Differences in formulation of PCZ have a significant impact on drug interactions, PK properties, and safety and tolerance of the antifungal. Currently, there are three FDA approved formulations that include the oral suspension, delayed-release tablets and injection for IV infusion. The oral suspension is indicated for the prophylaxis of invasive *Aspergillus* infections in patients who are immunocompromised. It is also approved for the treatment of oropharyngeal candidiasis, including cases that are refractory to ICZ and FCZ. The delayed-release tablets and injection, which became available on 2014, are indicated for the prophylaxis of invasive *Aspergillus* and *Candida* infections in severely immunocompromised patients. The FDA-approved indication for the delayed-release tablets and injection is based on PK and safety trials [2,83,84,85,86]. On the other hand, more supporting data on the oral suspension formulation of PCZ, including PK, safety and efficacy trials, have allowed for the recommendations per IDSA guidelines for additional indications, such as treatment of invasive infections of aspergillosis, allergic bronchopulmonary aspergillosis, salvage consolidation therapy in meningeal cryptococcosis relapse, and pulmonary cryptococcosis in patients who are not immunocompromised [87,88,89].

The extent to which drug interactions can occur is also dependent on the different formulations of PCZ. While the bioavailability of the oral suspension is highly affected by the concomitant use of medications that affect gastric pH and motility, such as H2RA antagonists, PPIs, and metoclopramide, the delayed-release tablets were not significantly impacted by the concomitant use of these drugs [90]. This also applies to the IV injection which is unaffected by drug interactions by default since it bypasses first-pass metabolism. In terms of PK properties, the delayed-release tablets have more predictable bioavailability and higher serum concentrations [91]. However, they cannot be crushed or chewed, removing them as an option for critically-ill patients, or those unable to swallow; this is where the IV injection formulation plays a major role [91]. In general, the tablet formulation has less inter-patient variability, better bioavailability which allows for once-daily administration (*versus* four times daily administration with the oral suspension), and absorption that is not affected by changes in gastric pH or motility, as previously mentioned [92]. Additionally, the tablets can be taken without regards to meals, and they have higher and more consistent mean serum concentrations compared to the oral suspension. Consequently, using the tablet formulation can potentially optimize treatment efficacy in IFIs. [92]. The tablet and injection formulations have shown a slightly similar safety profile to that of the oral suspension and are generally well tolerated. The delayed-released tablets have shown to cause diarrhea, pyrexia, nausea and hypoglycemia; while the IV injection has shown to cause diarrhea, hypokalemia, pyrexia, and nausea [2]. In clinical trials, the IV injection has also shown to cause thrombophlebitis when multiple peripheral infusions are given through the same vein [2].

## 58. Summary

PCZ is an extended-spectrum antifungal agent with antifungal activity against yeasts, filamentous fungi and azole resistant *Candida* species. PCZ exerts its antifungal activity by inhibiting fungal cell wall synthesis. It is orally bioavailable and is absorbed through the GI tract independent of pH. The systemic exposure of PCZ is optimized when it is taken with food or a nutritional supplement. Nevertheless, this only applies to the suspension formulation since the bioavailability of PCZ tablet and injection formulations is unaffected by the presence of food. PCZ has a large volume of distribution and is highly protein bound. The antifungal agent is metabolsed in the liver to several inactive glucuronide metabolites through the UGT enzyme pathway. The major elimination route of PCZ and its metabolites is fecal (~77%) and only small fractions are detected in the urine (~14%). The half-life of PCZ is long (>24 h) which allows this drug to be administered once daily. However, due to its unique oral absorption profile, PCZ is best administered as divided doses with a maximum daily dose of 800 mg. Age, gender, race and diseases, such as persistent febrile neutropenia and refractory IFIs, do not appear to alter the PK of PCZ. Dose adjustments are not needed in patients with renal failure and patients who are on hemodialysis. Furthermore, anecdotal PK, efficacy and safety data support the use of PCZ as salvage therapy in pediatric patients with IFIs. The use of PCZ is contraindicated in pregnancy and lactation. In adults, PCZ is efficacious, safe and well tolerated in the area of oropharyngeal candidiasis associated with HIV/AIDS infection, prophylaxis in neutropenia secondary to chemotherapy or in severe graft-*versus*-host disease associated with hematopoietic stem-cell transplantation. The most common adverse effects of PCZ include headache, fatigue, nausea and dry mouth. Potential drug-drug interactions exist between PCZ and other therapeutic agents including cimetidine, proton pump inhibitors, cyclosporine, tacrolimus, phenytoin, and rifabutin through either hepatic enzyme inhibition (CYP3A4) or induction. However, the potential for drug interactions is highly dependent on the formulation of PCZ, especially when coadministered with medications that affect gastric pH and motility, as the tablet and injection formulations have shown to be unaffected. Drug interactions also exist between PCZ and antiretrovirals used for the treatment of HIV, such as atazanavir, ritonavir, efavirenz, and fosamprenavir. Further research is needed in order to establish drug interactions between PCZ and other NRTIs, NNRTIs and PIs. Formulary limitation warrants PCZ oral administration in immunocompromised patients with functional GI tracts who fail conventional antifungal therapies or who are suspected to have a breakthrough fungal infection. Although a comparative study demonstrated that most patients with compromised gastrointestinal function can still achieve a therapeutic serum concentration of PCZ, future studies are warranted in order to confirm these results. Nevertheless, the new IV injection formulation is an attractive option for this patient population and will play a significant role when patients are unable to receive oral formulations of PCZ. Published studies present with several limitations in their study designs, including their retrospective nature, small sample sizes, descriptive analysis of the data, and lack of assessment of compliance of concomitant medications. This provides ample room for future research with better-controlled clinical trials in order to establish the role of PCZ in the treatment and prevention of several fungal infections, and potentially expand its FDA indications and role in clinical practice.
